# *Arabidopsis* ERF1 Mediates Cross-Talk between Ethylene and Auxin Biosynthesis during Primary Root Elongation by Regulating *ASA1* Expression

**DOI:** 10.1371/journal.pgen.1005760

**Published:** 2016-01-08

**Authors:** Jie-Li Mao, Zi-Qing Miao, Zhen Wang, Lin-Hui Yu, Xiao-Teng Cai, Cheng-Bin Xiang

**Affiliations:** 1 School of Life Sciences, University of Science and Technology of China, Hefei, Anhui Province, China; 2 Hefei National Laboratory for Physical Sciences at the Microscale, University of Science and Technology of China, Hefei, Anhui Province, China; Peking University, CHINA

## Abstract

The gaseous phytohormone ethylene participates in the regulation of root growth and development in *Arabidopsis*. It is known that root growth inhibition by ethylene involves auxin, which is partially mediated by the action of the *WEAK ETHYLENE INSENSITIVE2/ANTHRANILATE SYNTHASE α1* (*WEI2/ASA1*), encoding a rate-limiting enzyme in tryptophan (Trp) biosynthesis, from which auxin is derived. However, the molecular mechanism by which ethylene decreases root growth via *ASA1* is not understood. Here we report that the ethylene-responsive AP2 transcription factor, ETHYLENE RESPONSE FACTOR1 (ERF1), plays an important role in primary root elongation of *Arabidopsis*. Using loss- and gain-of-function transgenic lines as well as biochemical analysis, we demonstrate that ERF1 can directly up-regulate *ASA1* by binding to its promoter, leading to auxin accumulation and ethylene-induced inhibition of root growth. This discloses one mechanism linking ethylene signaling and auxin biosynthesis in *Arabidopsis* roots.

## Author Summary

Ethylene is a gaseous phytohormone that plays critical roles in plant development and defense. It is well known that ethylene inhibits primary root elongation through effects on auxin. However, it is not clear how ethylene signal is translated into auxin. In this report, the highly ethylene-responsive transcription factor ETHYLENE RESPONSE FACTOR1 (ERF1) is demonstrated to positively regulate *ASA1*, encoding *ANTHRANILATE SYNTHASE α1*, a rate-limiting enzyme in Trp biosynthesis where auxin is derived, by directly binding to its promoter and activating *ASA1*. Consequently, auxin biosynthesis is promoted, leading to increased auxin accumulation and thus inhibition of primary root elongation. This study unravels a molecular mechanism that bridges ethylene signaling and auxin biosynthesis in primary root elongation.

## Introduction

Phytohormones are central regulators of plant root growth and development. Each root development process is determined by a network of interacting signals to give the final architecture of the root[1]. Ethylene and auxin have been shown to regulate some of the same developmental processes, including primary root elongation[2–4]. Although the crosstalk between ethylene and auxin in regulating primary root elongation is well characterized[3,5–9], there is still a significant lack of understanding of the molecular mechanism that links ethylene signaling and auxin biosynthesis.

Ethylene, a gaseous plant hormone, acts as a key regulatory signal during the plant life cycle[10,11]. The biosynthesis of ethylene begins from methionine, which is converted to *S*-adenosyl-methionine (SAM) by SAM synthetase. Then a family of 1-aminocyclopropane-1-carboxylic acid (ACC) synthases (ACS) converts SAM to ACC. This reaction is a rate-limiting step and a key regulatory point in ethylene biosynthesis[12]. Finally, ACC is converted to ethylene by ACC oxidase (ACO)[13,14]. In the *Arabidopsis* genome, there are 12 members in *ACS* gene family, which display overlapping temporal and spatial expression patterns and are responsive to a variety of biotic and abiotic stresses and hormones, such as auxin[15,16]. Apparently all major components of ethylene signal transduction have been identified by the successful isolation of a series of ethylene response mutants and a precise ethylene signaling pathway has been established[17–21]. Once ethylene is synthesized, it is perceived by any of five membrane bound protein receptors ETHYLENE RESPONSE1 (ETR1), ETR2, ETHYLENERESPONSE SENSOR1 (ERS1), ERS2, and ETHYLENEINSENSITIVE4 (EIN4), which possess sequence similarity to bacterial two-component His kinases[22–24]. The binding of ethylene to its receptor results in inhibition of a Raf-like Ser/Thr protein kinase CONSTITUTIVE TRIPLE RESPONSE1(CTR1)[25]. Inhibited CTR1 loses its ability to phosphorylate and repress a positive component of the ethylene signal pathway, the membrane protein ETHYLENE INSENSITIVE2 (EIN2)[26]. The active form of EIN2 stabilises the transcription factors of the EIN3 family located in the nucleus. The EIN3 proteins subsequently bind to the promoters of the *ERF* genes and activate their transcription[27,28]. Thus a transcriptional cascade commencing with the sensing of ethylene is triggered to produce the ethylene response.

ERFs, which contain an AP2 DNA-binding domain, form a plant-specific superfamily of 122 transcriptional factors in *Arabidopsis*[29]. ERFs influence a variety of functions involved in plant development and also play important roles in response to biotic and abiotic stresses[30–34], through specifically binding to sequences containing GCCGCC motifs (GCC-box) in the regulatory region of downstream genes[35]. It was reported that GCC-box is not well conserved in ethylene responsive genes, suggesting that other types of transcription factors may also be activated by EIN3 and involve in transcriptional cascade caused by ethylene[7]. *ERF1* (AT3G23240) is a downstream component of the ethylene signaling pathway and is directly regulated by EIN3 at the transcriptional level[27]. It is well known that *ERF1* is a key integrator of the jasmonic acid (JA) and ethylene signaling pathways involved in the regulation of defence response genes such as *b-CHI* and *PDF1*.*2*[36]. Ethylene signaling is also involved in plant responses to both salt and water stress, as ethylene insensitive mutants were more salt sensitive[37–39]. *ERF1* also plays a positive role in abiotic stress responses such as salt, drought, and heat stress[40]. In addition to responding to biotic and abiotic stress, *ERF1* further mediates ethylene responses in developmental processes, such as the inhibition of primary root growth and hypocotyl elongation in the dark. This has been confirmed by the production of transgenic plants with constitutively activated *ERF1*, which displayed phenotypes similar to that are observed in *ctr1* mutant, *EIN3*-overexpressing plants, and wild-type plants treated with ethylene[25,27,41]. Recently, *ERF109* was shown to mediate crosstalk between JA signaling and auxin biosynthesis[42].

Root growth relies on two essential developmental processes: cell division in the root meristem and elongation of cells produced by the root meristem[43]. Root cell elongation can be affected by diverse endogenous and exogenous factors such as ethylene[3], auxin[44], and calcium[45]. Ethylene, and its precursor ACC, reduces root elongation in a concentration-dependent manner by inhibition of the cell elongation process[4,6]. The crosstalk between ethylene and auxin has been well investigated[3,6]. The most interesting discovery for auxin/ethylene crosstalk in recent years is that *Arabidopsis* pyridoxal-phosphate -dependent aminotransferase, VAS1, uses the ethylene biosynthetic intermediate methionine as an amino donor and the auxin biosynthetic intermediate indole-3-pyruvic acid as an amino acceptor to produce L-tryptophan and 2-oxo-4-methylthiobutyric acid[46]. Many mutants that affect auxin synthesis, distribution, or signaling also result in abnormal responses to ethylene[8,47–50], such as, mutants of *AUX1* and *EIR1/AGR/PIN2* involved in auxin transport, *AXR2*/*IAA7* and *AXR3*/*IAA17* in the auxin signal pathway, or the auxin receptor *TIR1*, which all exhibit ethylene-insensitive root growth[5,20,49,51–53]. Auxin biosynthetic genes encoding enzymes such as *WEI2/ ANTHRANILATE SYNTHASE α1* (*ASA1*), *WEI7/ASB1*, *TAA1*, and *TAR1*, which are regulated by ethylene, also exhibit ethylene-insensitive root growth[8,9]. *YUC* genes also play an important role in root responses to ethylene[54]. These studies suggest that the inhibition of primary root growth caused by ethylene requires auxin biosynthesis, transport, or signaling. *WEI2* encodes the *α*-subunit of the enzyme anthranilate synthase in Trp-dependent auxin biosynthesis. Its expression in roots can be induced by ethylene and *wei2* mutations cause ethylene-insensitive root growth phenotypes[8]. In *Arabidopsis* roots, ethylene promotes auxin biosynthesis in a *ASA1*-dependent manner[8], this is an important molecular mechanism by which ethylene exerts its effect on promoting auxin biosynthesis. However, the molecular mechanism for regulation of *ASA1* by ethylene is not well understood.

Here, we report that ERF1, a downstream AP2 transcription factor in the ethylene signaling pathway, positively regulates auxin biosynthesis during inhibition of ethylene-mediated primary root growth. Transgenic plants with constitutive expression or knockdown of *ERF1* displayed similar root development phenotype to mutants of ethylene signaling. ERF1 affected auxin accumulation through directly binding to the *ASA1* promoter and positively regulating *ASA1* expression. Our results indicate that ERF1 plays a pivotal role in the inhibition of ethylene-induced primary root growth in *Arabidopsis* and acts as the crosstalk node between ethylene and auxin in primary root elongation.

## Results

### The expression of *ERF1* is responsive to ethylene and dependent on ethylene signaling

**Fig 1:**
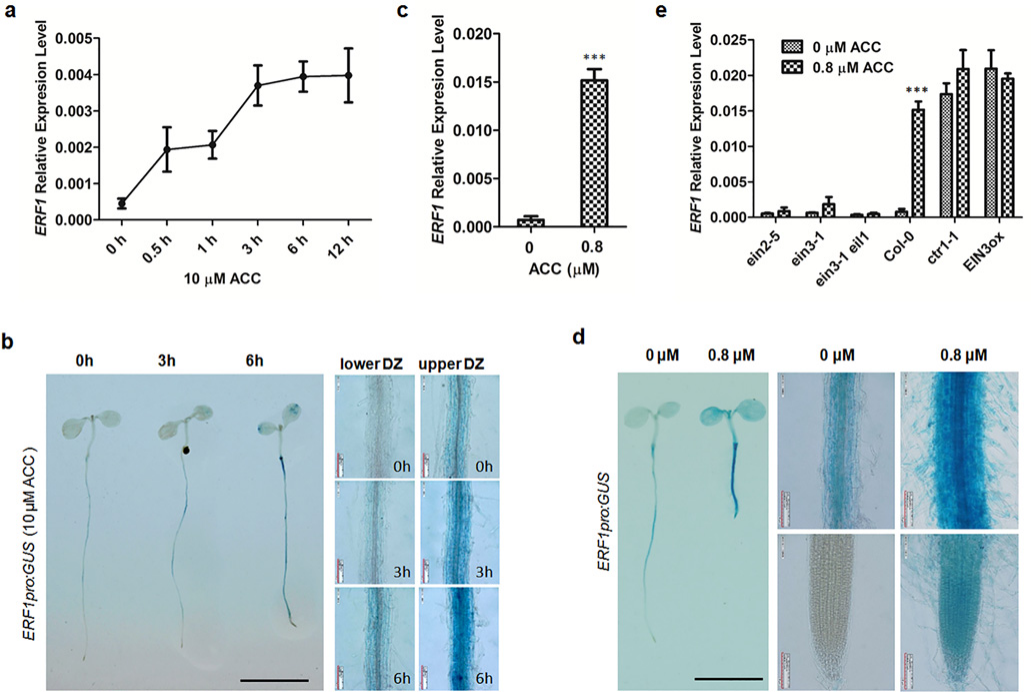
*ERF1* expression is responsive to ethylene. (**a**) Ethylene-induced *ERF1* expression in wildtype. Seeds of Col-0 were germinated on MS medium for 5 d then treated with 10 μM ACC for 0, 0.5, 1, 3, 6, and 12 h. The transcriptional level of *ERF1* was detected by quantitative RT-PCR (qRT-PCR). Values are mean ± SD of three replicates. ACC, 1-aminocyclopropane-1-carboxylic acid (precursor of ethylene biosynthesis). (**b**) Ethylene-induced expression of *ERF1pro*:*GUS*. Five-day-old seedlings of transgenic lines of were treated with 10 μM ACC for 0, 3, and 6 h before GUS staining. Upper DZ and lower DZ represent different primary root regions. Scale bar, 0.5 cm. (**c**) Ethylene-induced expression of *ERF1* in wildtype. Seeds of Col-0 were germinated on MS medium with 0 or 0.8 μM ACC for 5 d, and relative *ERF1* transcription levels measured by qRT-PCR. Values are mean ± SD of three replicates (***P<0.001). Asterisks indicate Student’s t-test significant differences. (**d**) Ethylene-activated expression in *ERF1*_*pro*_:*GUS* lines. Transgenic plants were grown MS medium with either 0 or 0.8 μM ACC for 5 d before GUS staining assay. Scale bar, 0.5 cm. (**e**) The relative *ERF1* expression level was determined in ethylene signaling related mutants *ein2-5*, *ein3-1*, *ein3-1eil1*and compared to wildtype (Col-0) seedlings. Seedlings carrying constructs for either constitutive (*ctr1-1*) or inducible *EIN3-FLAG* (*iE*/*qm*) (*EIN3ox*) expression were also examined. Seedlings were geminated on MS medium with either 0 or 0.8 μM ACC for 5 d. Seeds of *EIN3ox* were grown on medium containing 1 μM β-estradiol and 0 or 0.8 μM ACC. Roots of seedlings were used for qRT-PCR analysis. Values are mean ± SD of three replicas (***P<0.001). Asterisks indicate Student’s t-test significant differences.

To study the role of *ERF1* in ethylene response, we examined the ethylene-induced expression of *ERF1* in 5-day-old wildtype seedlings (Col-0) grown on Murashige and Skoog (MS) medium with 10 μM ACC for 0–12 h. The *ERF1* transcript level in whole seedlings was measured by quantitative real-time PCR (qRT-PCR). Consistent with previous studies[36,40], the *ERF1* transcript level was rapidly induced by ACC treatment. *ERF1* increased in a biphasic manner showing a 4-fold increase after 0.5 h, and a second plateau increasing by approximately 8-fold at 3 h. Thereafter, the *ERF1* transcript levels remained high (Fig 1A).

The spatial expression pattern of *ERF1*, was determined by assaying *ß*-glucuronidase (GUS) activity of transgenic plants carrying an *ERF1*_*pro*_:*GUS* construct, in which a GUS reporter gene was under the control of the *ERF1* promoter (*ERF1*_*pro*_, a 3.0kb promoter fragment). *ERF1* was mainly expressed in the maturation zone of the primary root of seedlings (Fig 1B). To examine the response of *ERF1* to ethylene, we treated 5-day-old *ERF1*_*pro*_:*GUS* seedlings with 10 μM ACC for 0–6 h and found that the expression of *ERF1* was induced in the maturation zone and cotyledons as the ACC treatment time increased. Stronger induction was observed in the upper maturation zone (Fig 1B).

To determine the long-term response of *ERF1* to ethylene, we grew wildtype seedlings on MS medium with either 0 or 0.8 μM ACC for 5 d and examined the relative transcript levels. The abundance of *ERF1* increased about 21 times by ACC treatment compared with the 0 μM ACC control (Fig 1C). Transgenic seeds carrying *ERF1*_*pro*_:*GUS* were directly germinated on MS supplemented with or without 0.8 μM ACC for 5 d before GUS analysis. Strikingly in ACC-supplemented seedlings, GUS staining increased along the entire root and with weaker staining was also visible in the primary root tip compared to the 0 μM ACC control (Fig 1D).

GUS staining of *ERF1*_*pro*_:*GUS* transgenic lines showed that *ERF1* expression was mainly present in roots and old leaves (S1A–S1E Fig). ERF1 nuclear-specific subcellular localization was demonstrated in transgenic plants carrying a *CaMV 35S*-driven *ERF1* construct fused to *GFP* (S1F Fig). These results suggest that ERF1 may play a role in root development in response to ethylene signaling.

To confirm ethylene-dependent induction of *ERF1* expression, we analysed the *ERF1* transcript level in the ethylene signaling mutants *ein2-5*, *ein3-1*, *ein3-1eil1* and compared them to wildtype seedlings. *ERF1* transcription was also measured in lines of constitutive or *β*-estradiol inducible expression, *ctr1-1* and *EIN3-FLAG* (*iE*/*qm*) (*EIN3ox*), respectively. Expression of *ERF1* could not be induced by ACC in the *ein2-5*, *ein3-1* and *ein3-1eil1* mutants, while it was constitutively expressed in the *ctr1-1* and *EIN3ox* seedlings (Fig 1E). These results suggested that ethylene-induced expression of *ERF1* is dependent on the ethylene signaling pathway.

### Genetic analysis of *ERF1* in primary root elongation

**Fig 2:**
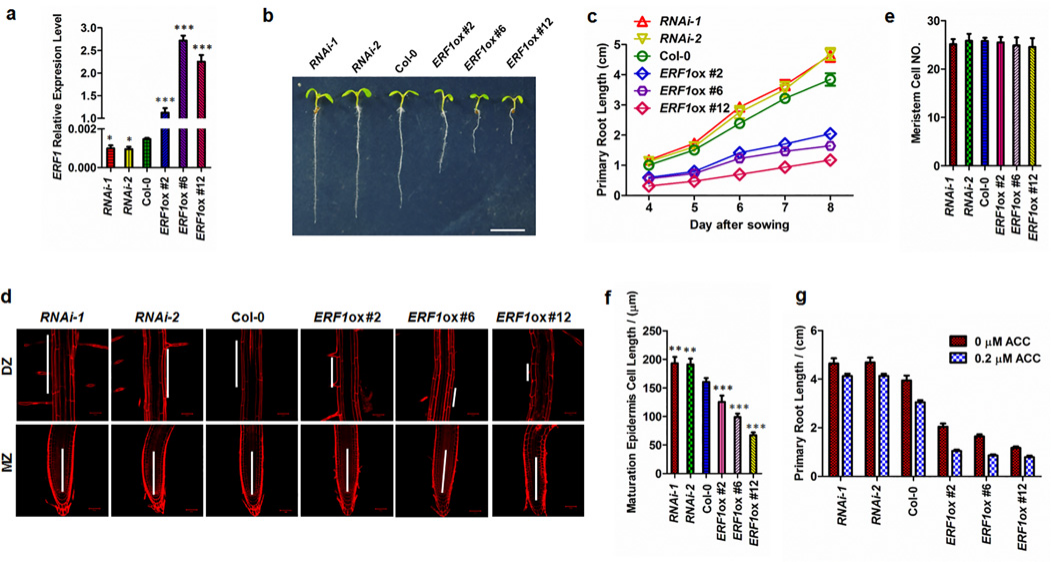
Role of ERF1 in ethylene-mediated inhibition of primary root elongation. (**a-c**) Primary root phenotypes of *ERF1* knockdown (*RNAi-1*, *RNAi-2*) and overexpression (*ERF1ox #2*, *ERF1ox #6*, *ERF1ox #12*) transgenic lines. The expression level of *ERF1* in 5 day-old-seedlings was tested by qRT-PCR (**a**). Values are mean ± SD of three replicas (*P<0.05, ***P<0.001). Asterisks indicate Student’s t-test significant differences. The representative seedlings were photographed (**b**). Scale bar, 1 cm. The primary root length of these related transgenic lines and Col-0 was measured from 4 to 8 d (**c**). Data shown are average and SD (Values are mean±SD, n = 20). (**d-f**) Epidermal cell length within the DZ was affected in *ERF1* knockdown (*RNAi-1*, *RNAi-2*) and overexpression (*ERF1ox #2*, *ERF1ox #6*, *ERF1ox #12*) transgenic lines. The primary root MZ and DZ of the 5-day-old seedlings of indicated lines were photographed. Representative images are shown (**d**). Root meristem cell number (**e**) and epidermal cell length of the maturation zone (**f**) were measured. The root meristem cell number was counted from the QC to the first elongation cell in the cortex file. The mean ± SD (n = 20) is shown, **P<0.01, ***P<0.001). Asterisks indicate Student’s t-test significant differences. (**g**) Primary root length of *ERF1* knockdown (*RNAi-1*, *RNAi-2*) and overexpression lines grown on medium with or without ACC treatment. Five-day-old seedlings grown on MS medium were transferred to MS medium containing 0 or 0.2 μM ACC and growth vertically for 3 d. The mean ± SD is shown (n = 50).

To investigate the function of *ERF1* in *Arabidopsis* root growth, we generated transgenic knockdown and overexpression lines. The phenotype of these plants was confirmed with the analysis of *ERF1* expression (S2 Fig). From three overexpression lines and two RNAi knockdown lines (Fig 2A), we found that the primary roots of the lines overexpressing *ERF1* (*ERF1ox*) were stunted. In contrast, the RNAi lines showed longer primary roots compared to the wildtype controls (Fig 2B and 2C). Similar results of root elongation were observed in etiolated seedlings (S3 Fig). Root elongation in *ERF1ox* lines was similar to mutants with an activated ethylene signal pathway, while root elongation in *ERF1* RNAi lines was similar to mutants with defects in ethylene signal pathway (S4A Fig).

Ethylene signaling mutants *ein2-5*, *ein3-1*, *ein3-1eil1*, *ctr1-1*, and *EIN3ox* also display abnormal primary root development with or without ACC treatment[25,26,55–58]. The roots of *ein2-5*, *ein3-1* and *ein3-1eil1* seedlings, in which the corresponding genes positively regulate the ethylene signal pathway, were longer and more insensitive to ACC compared to wildtype (S4A Fig). However, the roots of *ctr1-1* and *EIN3ox*, in which ethylene signaling was significantly active, were shorter than wildtype roots.

To explore the primary root elongation of the *ERF1*-related transgenic lines, we investigated the primary root in three developmental zones, the differentiation zone (DZ, also known as maturation zone), the elongation zone (EZ), and the meristem zone (MZ). Root meristem size was measured as the cell number from the quiescent centre (QC) to the first elongated cell in the cortex[59]. No significant change in meristem cell number was observed among the transgenic lines compared to the wildtype (Fig 2D and 2E). However, cell length had dramatically decreased in the DZ of *ERF1ox* lines, whereas it was greater in the DZ of the RNAi lines compared to wildtype (Fig 2D and 2F). These results suggest that *ERF1* contributes to root length via altering cell elongation but not cell division (Fig 2D–2F), which is a similar effect to that produced by exogenously applied auxin[60]. This agrees with the root inhibition mechanism resulting from ACC treatment and the ethylene signal pathway mutants, *ein2-5* and *ctr1-1*[3,6,61].

Since *ERF1* is downstream of the ethylene signal pathway, we proposed that the inhibition of root elongation by ethylene might be *ERF1*-dependent. To analyze this, wildtype, *ERF1ox*, and RNAi lines were grown on MS plates for 5 d, then transferred to plates with or without ACC for 3 d, and primary root lengths were measured. We found that the primary root lengths of the RNAi lines were greater than controls, but those of *ERF1ox* lines were shorter than controls (Fig 2G). Furthermore, the difference between *ERF1* expression levels in *ERF1* RNAi lines and wildtype was augmented by ACC treatment (S4B Fig). Taken together, these results suggest that ethylene inhibits primary root elongation mainly through *ERF1*. ERF1, as a positive regulator of ethylene signaling, appears to play a negative role in regulating root cell elongation, leading to dramatically shortened primary roots under conditions of hyperactive ethylene signaling.

### ERF1 enhances auxin accumulation in roots

**Fig 3:**
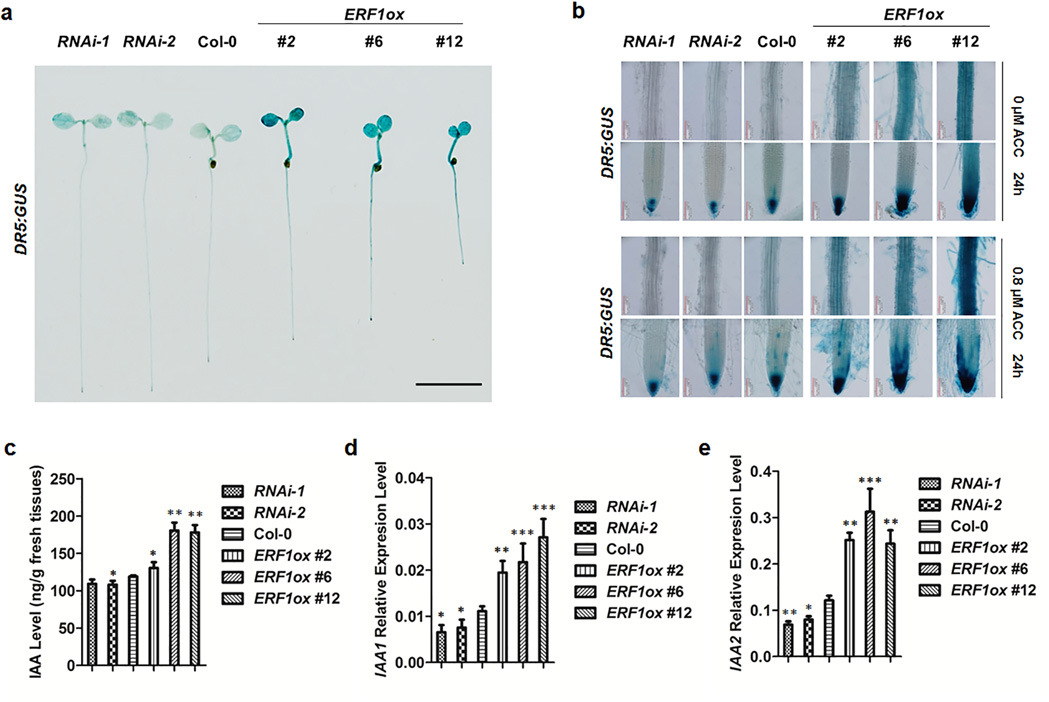
ERF1 enhances auxin accumulation in roots. (**a**) *DR5*:*GUS* expression in 5-day-old seedlings of Col-0,*ERF1* knockdown (*RNAi-1*, *RNAi-2*) and overexpression lines (*ERF1ox #2*, *ERF1ox #6*, *ERF1ox #12*). Three independent experiments were done, and each replica containing 15 plants for each line. Representative seedlings were photographed. Scale bar, 0.5 cm. (**b**) *DR5*:*GUS* expression in maturation region and root tip of primary root. Five-day-old seedlings of Col-0, *ERF1* knock-down (*RNAi-1*, *RNAi-2*) and over-expression (*ERF1ox #2*, *ERF1ox #6*, *ERF1ox #12*) lines were treated with 0 or 0.8 μM ACC for 24 h before GUS activity was assayed. More than 20 plants were observed for each line. Representative photos were displayed. (**c**) Free IAA content. Seeds of *ERF1* overexpression and RNAi lines were grown on MS plates for 5 d before root IAA content was measured. The mean ± SD of three replicas is shown (*P<0.05, **P<0.01). Asterisks indicate Student’s t-test significant differences. (**d-e**) The transcript level of *IAA1* and *IAA2*. Seeds of *ERF1* overexpression and RNAi lines were grown on MS plates for 5 d before RNA isolation from the roots. Transcript abundance of *IAA1* and *IAA2* was measured using qRT-PCR. The mean ± SD of three replicates is shown (*P<0.05, **P<0.01, ***P<0.001). Asterisks indicate Student’s t-test significant differences.

The reduced primary root elongation phenotype in *ERF1* overexpression lines was similar to wildtype plants grown on the medium containing auxin[6], consistent with the proposal that ethylene enhances auxin biosynthesis to inhibits root elongation [3,6]. To study whether ERF1 enhances auxin accumulation in *Arabidopsis* roots, Firstly, we introduced a *DR5*:*GUS* reporter, an auxin reporter responding to endogenous auxin[62], into *ERF1* knockdown and overexpression lines (Fig 3A and S5A Fig). Expression of *DR5*:*GUS* in primary roots, including the MZ and DZ was significantly increased in the *ERF1* overexpression background. GUS expression occurred even in the absence of added ACC, in the root as well as in the cotyledons and hypocotyl (Fig 3A and 3B).The distribution of GUS staining in the primary root tip extended to other tissues compared to wildtype, i.e., also occurring in the epidermal cells of the MZ (Fig 3B, #12). In addition, *DR5*:*GUS* expression in DZ occurred not only in the xylem as for wildtype, but also in epidermis, cortex and endodermal tissues (Fig 3B, #2, #6, and #12). In contrast, the GUS activity decreased in the primary root tip and stele in *ERF1* knockdown background compared with wildtype (Fig 3B, *RNAi-1* and *-2* compared with Col-0, respectively). The difference of *DR5*:*GUS* expression in these lines were more evident when treated with ACC for 24 h (Fig 3B). Moreover, the IAA content was higher in the *ERF1* overexpression lines and lower in the RNAi lines compared with wildtype (Fig 3C). In accordance with the increased auxin, the expression of *IAA1* and *IAA2*, which are auxin-responsive marker genes, was activated in overexpression lines of *ERF1* and down regulated in knockdown lines (Fig 3D and 3E). Our results indicate that, ERF1 restrains primary root elongation by increasing auxin.

### ERF1 directly binds to *ASA1* promoter region *in vitro* and *in vivo*

Ethylene positively regulates the transcription of *ASA1* to increase auxin and inhibit root elongation[8]. This finding prompted us to examine whether ERF1 functions as a direct regulator of *ASA1*. Previous studies showed that ERF1 binds to a specific *cis*-element (a GCC-box related sequence) upstream of its target genes, to regulate downstream gene expression[27,40].We analysed the promoter sequence of *ASA1* and found one GCC-box at 27 bp upstream of the translational start codon. We sought to test whether this GCC-box could provide a handle for ERF1 to regulate *ASA1* directly.

**Fig 4:**
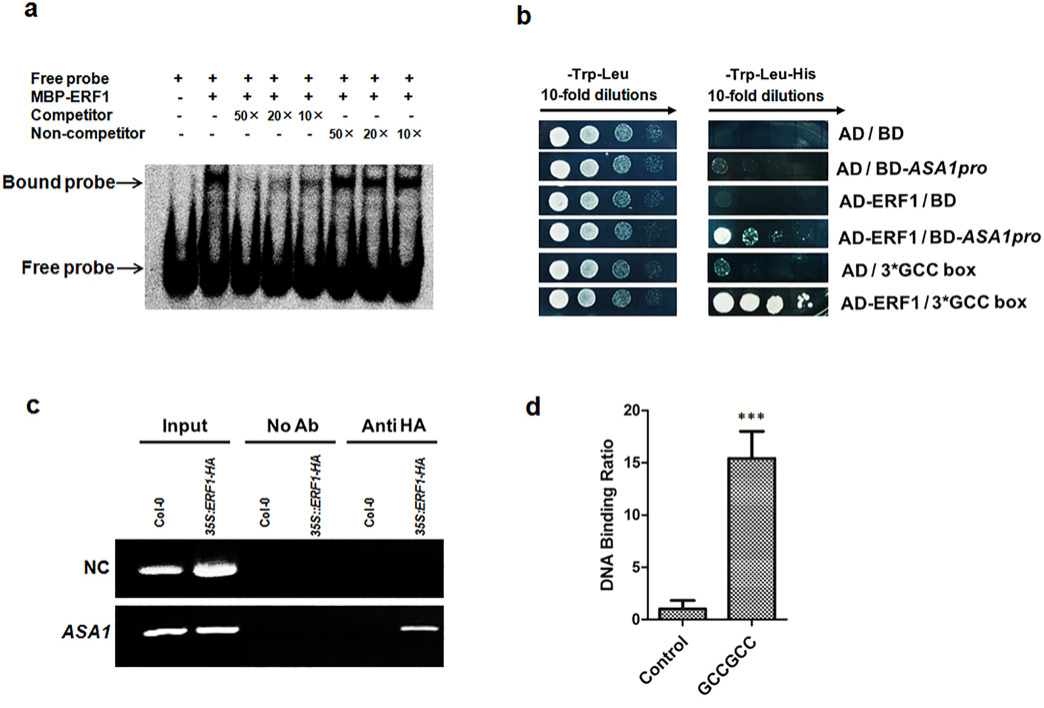
ERF1 directly binds to *ASA1* promoter region *in vitro* and *in vivo*. (**a**) EMSA assay for binding to GCC-box sequence in the promoter of *ASA1* by ERF1 protein *in vitro*. Dig-labelled probes were incubated with ERF1-MBP protein. As indicated, unlabelled probes were used as competitors, unlabelled probes with mutated GCC-box sequence were used as non-competitors, and the ERF1-MBP protein bound probers were separated from free probes by an acrylamide gel. (**b**) Yeast-one-hybrid assay. pGADT7/ERF1 (AD-ERF1) and pHIS2/ASA1pro (BD-ASA1pro) constructs were co-transformed into yeast strain Y187. AD-empty and BD-empty, AD-empty and BD-ASA1pro, AD-ERF1 and BD-empty, AD-empty and BD-3*GCC-box were used as negative controls while AD-ERF1 and BD-3*GCC-box were used as a positive control. (**c**) Chromatin immunoprecipitation-PCR for *ASA1* promoter. Roots of 5-day-old 3*5S*:*HA*:*ERF1* and Col-0 seedlings were used. Anti-HA antibodies were used for the enrichment of the DNA fragments containing GCC-box in the promoter of *ASA1*. The results were determined by real-time PCR. *Tub8* was used as a negative control (NC). (**d**) Quantitative real-time PCR was performed using the same ChIP products and PCR primers flanking GCC-boxes in *ASA1* promoter as in **c**. The region of *ASA1* that do not contain GCC-box was used as negative control. Values are mean ± SD of three replicas (***P<0.001). Asterisks indicate Student’s t-test significant differences.

To determine if there was a direct physical interaction of the ERF1 protein with the *ASA1* promoter sequence, we conducted an electrophoretic mobility shift assay (EMSA) with the full-length ERF1 protein fused to a maltose binding protein (MBP-ERF1) that was expressed in *E*. *coli* and purified through affinity chromatography. As shown in Fig 4A, the ERF1-MBP fusion protein was able to specifically bind digoxigenin-labelled DNA probes that contained the GCC-box motif of the *ASA1* promoter. Moreover, the binding specificity was confirmed by competition with native DNA probes, or probes carrying a mutated GCC-box. Unlabelled native DNA probe was used as a competitor and unlabelled promoter fragment containing the mutant form of the GCC-box motif as a non-competitor. The EMSA results showed that ERF1 specifically binds to the promoter sequence containing a GCC-box of *ASA1* promoter *in vitro*, but not to the DNA probe containing the mutant GCC-box sequence (Fig 4A). In addition, we carried out a yeast-one-hybrid assay that showed ERF1 was able to bind to the GCC-box sequence in the *ASA1* promoter in yeast cells (Fig 4B).

To confirm whether this specific binding occurs *in planta*, we generated transgenic *Arabidopsis* plants expressing the *35S* promoter-driven HA tagged *ERF1* construct (S6 Fig). Transgenic lines showing expected phenotypes were used for chromatin immunoprecipitation (ChIP) with anti-HA antibodies (Roche, USA). As shown in Fig 4C, the chromatin immunoprecipitated with anti-HA antibodies was significantly enriched for the *ASA1* promoter containing GCC-box fragments in the ChIP-PCR assay. This was further confirmed by quantitative real-time PCR performed using the same ChIP products and PCR primers flanking GCC-boxes in *ASA1* promoter (Fig 4D). These results suggest a specific binding of ERF1 to the promoter of *ASA1 in vivo*.

### ERF1 positively regulates the expression of *ASA1* in the roots

**Fig 5:**
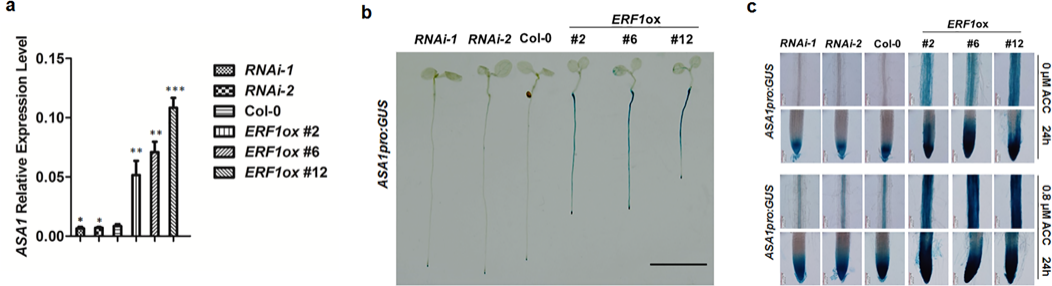
ERF1 up-regulates the expression of *ASA1* in roots. (**a**) Transcript levels of *ASA1* in 5-day-old Col-0, *ERF1* knockdown (*RNAi-1*, *RNAi-2*), and overexpression (*ERF1ox* #*2*, *ERF1ox* #*6*, *ERF1ox* #*12*) seedlings. Values are mean ± SD of three replicas (*P<0.05, **P<0.01, ***P<0.001). Asterisks indicate Student’s t-test significant differences. (**b**) *ASA1*_*pro*_:*GUS* expression in 5-day-old Col-0 and *ERF1* knockdown (*RNAi*-1, *RNAi*-2) and over-expression (*ERF1ox* #*2*, *ERF1ox* #*6*, *ERF1ox* #*12*) seedlings. Three independent experiments were done, and each replica containing 15 plants for each genotype. Representative seedlings were photographed. Scale bar, 0.5 cm. (**c**) *ASA1*_*pro*_:*GUS* expression in primary root maturation region and root tip of 5-day-old Col-0, *ERF1* knockdown (*RNAi-1*, *RNAi-2*), and overexpression (*ERF1ox* #*2*, *ERF1ox* #*6*, *ERF1ox* #*12*) seedlings without or with ACC treatment. More than 20 plants were observed for each genotype. Representative photos were displayed.

To investigate the consequence of ERF1 binding to the GCC-box of the *ASA1* promoter, we measured the *ASA1* expression level in *ERF1* transgenic lines by qRT-PCR. As predicted, the transcript level of *ASA1* in *ERF1* overexpression lines was significantly increased compared to wildtype without additional treatment. A small but statistically significant reduction of *ASA1* transcripts in *ERF1* knockdown lines was observed (Fig 5A). The difference in *ASA1* expression levels between knockdown lines of *ERF1* and wildtype was further increased by ACC treatment (S4C Fig). In addition, the increased expression level of *ASA1* in *35S*_*pro*_:*ERF1* plants agreed with the microarray results in a previous report that *ASA1* was upregulated in *35S*:*ERF1* transgenic plants[36]. These results suggest that *ASA1* is indeed a downstream target of ERF1.

To confirm further whether the expression level of *ASA1* was affected by ERF1, we introduced the *ASA1*_*pro*_:*GUS* reporter into *ERF1* knockdown and overexpression background and examined the primary root phenotype (Fig 5B and S5B Fig). Consistent with the *ASA1* expression level in *ERF1* transgenic lines (Fig 5A), we found that the intensity of GUS staining in *ASA1*_*pro*_:*GUS* significantly increased in an *ERF1* overexpression background and slightly reduced in the *ERF1* knockdown lines compared to wildtype (Fig 5C). Moreover, this difference was further exaggerated in response to ACC treatment (Fig 5C). Furthermore, the GUS staining patterns produced by *ASA1*_*pro*_:*GUS* in the *ERF1* knockdown lines was weaker than that of wildtype with ACC treatment. Taken together, these results suggest that ERF1 positively regulates *ASA1* expression in response to ethylene.

### The inhibition of primary root growth by ethylene is *ASA1*-dependent

**Fig 6:**
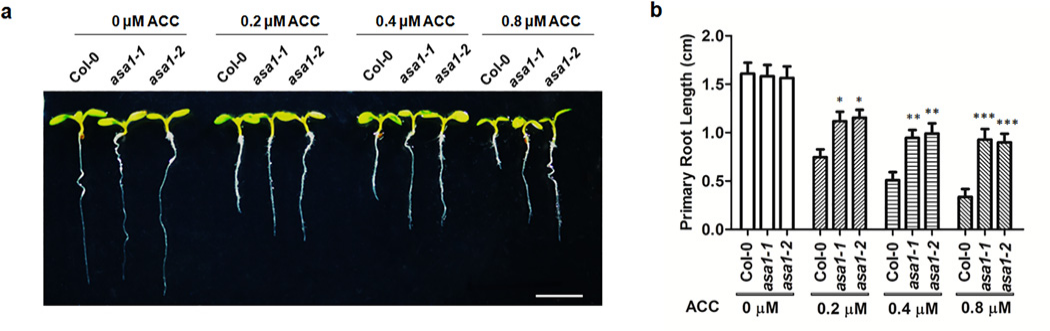
Root elongation of *asa1*mutants in response to ACC treatment. (**a**) Root elongation of Col-0 and *asa1* without and with ACC treatment. *CS16398* (*asa1-1*) and *CS16397* (*asa1-2*) are two different loss-of-*ASA1* mutants. Seeds of Col-0 and *asa1* were grown on MS plates without or with ACC for 5 d. Three independent experiments were displayed with similar results. Representative seedlings were photographed. Scale bar, 0.5 cm. (**b**) The primary root length of 5-day-old Col-0 and *asa1* mutants grown on medium without or with ACC. Values are mean ± SD of three replicas (*P<0.05, **P<0.01, ***P<0.001). Each replica contains 30 plants for each line. Asterisks indicate Student’s t-test significant differences.

Given that ethylene-inhibited root elongation involved auxin biosynthesis which is enhanced by ERF1 through regulating *ASA1* expression, we predicted that loss of *ASA1* would decrease the sensitivity to ethylene. To test this, we grew wildtype and *asa1* mutants (*asa1-1* and *asa1-2*) on MS medium with or without ACC. The *asa1* mutants grown on media containing different concentrations of ACC displayed longer primary roots than wildtype seedlings (Fig 6A and 6B). Our results support the notion that inhibition of root elongation by ethylene is *ASA1*-dependent. This observation is consistent with a previous finding that *asa1* mutants are insensitive to ethylene under dark conditions[8]. Furthermore, as shown in S7 Fig, *ERF1-RNAi* lines, *asa1*, and *ein2*-5 showed reduced response to ACC with respect to the effect of the ethylene on primary root growth compared to Col-0. For the reduced sensitivity of root to ACC, *asa1* and *ein2* were more evident than *ERF1-RNAi* lines, which might be due to the limitation of *ERF1-RNAi* materials, and/or some other factors participating in this process.

**Fig 7:**
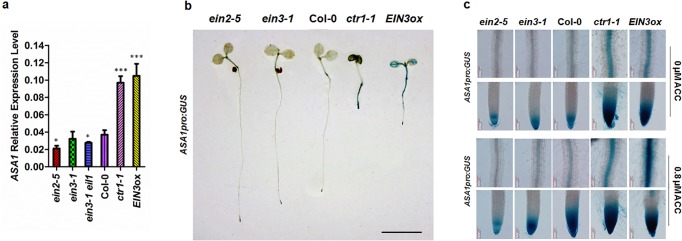
The expression of *ASA1* was regulated by ethylene signal pathway. (**a**) The expression of *ASA1* in ethylene signal pathway mutants. Ethylene signal pathway-related mutants *ein2-5*, *ein3-1*, *ein3-1eil1*, *ctr1-1*, and Col-0 were grown on MS medium for 5 d while the seeds of *EIN3-FLAG* (*iE*/*qm*) (*EIN3ox*) were grown on MS medium supplementing with 1 μM β-estradiol for 5 d. Total RNA was extracted from roots. The expression level of *ASA1* was checked by qRT-PCR. Values are mean ± SD of three replicas (*P<0.05, **P<0.01, ***P<0.001. Asterisks indicate Student’s t-test significant differences). (**b**) *ASA1*_*pro*_:*GUS* expression in 5-day-old seedlings. Three independent experiments were performed with each replica containing 15 plants for each line. Representative seedlings were photographed. Scale bar, 0.5 cm. (**c**) *ASA1*_*pro*_:*GUS* expression in primary root maturation region and root tip of 5-day-old seedlings without or with ACC treatment. More than 20 plants were observed for each genotype. Representative photos were displayed.

The primary root elongation of mutants (*ein2-5*, *ein3-1eil1* and *ein3-1*) was less sensitive to ACC compared to wildtype. The mutants with enhanced ethylene signals (*ctr1-1*, *EIN3ox*) showed dramatically shortened primary roots under normal conditions, mimicking wildtype seedlings treated with ACC[25,26] (S4A Fig). Since *ASA1* functions downstream of CTR1[8], we measured the *ASA1* transcript levels in these mutants in normal conditions and found that the expression level of *ASA1* in *ein2-5*, *ein3-1eil1* and *ein3-1* was lower than that of wildtype and conversely higher in *ctr1-1* and *EIN3ox* lines (Fig 7A). To confirm this further, we introduced *ASA1*_*pro*_:*GUS* reporter into *ein2-5*, *ein3-1*, *ctr1-1*, and *EIN3ox* backgrounds and found that the GUS staining pattern closely correlated with the qRT-PCR results (Fig 7B and 7C). Strong GUS activity in the root and cotyledons was observed in *ctr1-1* and *EIN3ox* backgrounds (Fig 7B). These results indicated that *ASA1* is downstream of these ethylene signal pathway components.

**Fig 8:**
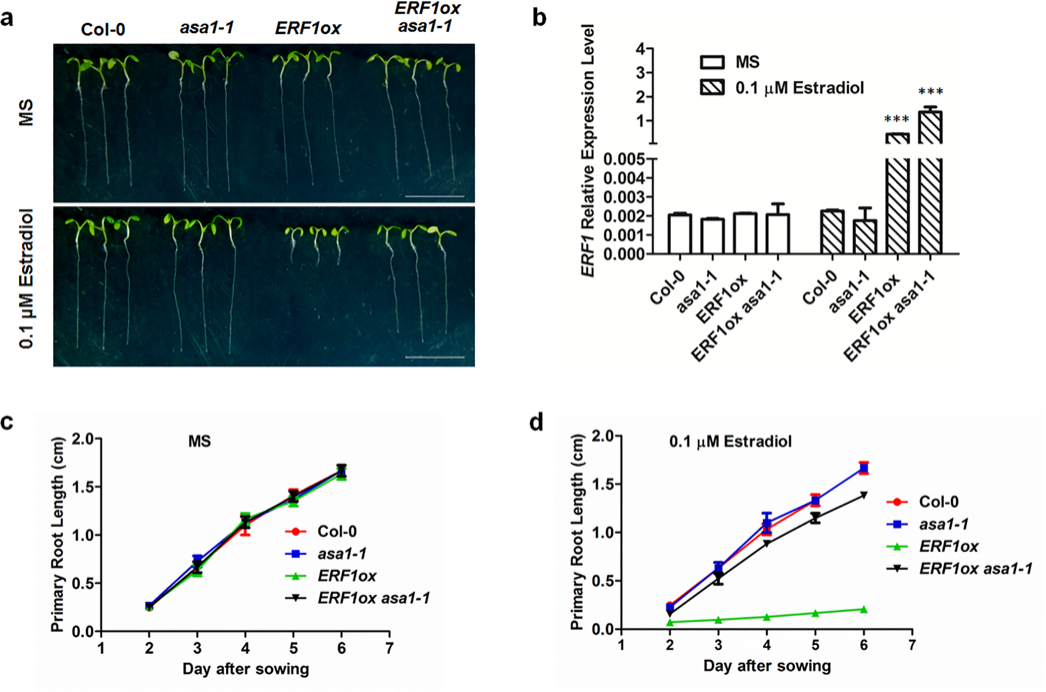
*ASA1* acts downstream of ERF1. (**a**) The primary root phenotypes of Col-0, *asa1-1*, *ERF1ox* and *ERF1ox asa1-1* seedlings grown on MS medium with either 0 or 0.1 μM estradiol for 5 d. *ERF1ox* is the transgenic plants expressing ERF1 protein under control of the estradiol-inducible promoter in Col-0 background. Scale bar, 1 cm. (**b**) qRT–PCR analysis of transcriptions of *ERF1*. The roots of 5-day-old Col-0, *asa1-1*, *ERF1ox* and *ERF1ox asa1-1* seedlings grown on MS medium with either 0 or 0.1 μM estradiol were used. Values are mean ± SD of three replicas (***P<0.001. Asterisks indicate Student’s t-test significant differences). (**c-d**) Primary root length of Col-0, *asa1-1*, *ERF1ox* and *ERF1ox asa1-1* seedlings grown on MS medium with either 0 or 0.1 μM estradiol were measured at the fifth days. Data shown are average and SD (Values are mean ± SD, n = 20).

To confirm this genetically, we introduced an estradiol-inducible *ERF1* overexpression in *asa1* mutant background (*ERF1ox asa1-1*). When *ERF1* is overexpressed, the primary root of *ERF1ox asa1-1* is significantly longer than that of *ERF1ox* and a little shorter than Col-0 (Fig 8, S8 Fig). The result demonstrates that *ASA1* is downstream of ERF1, and suggests that ethylene-inducible *ERF1* controls root elongation through regulating *ASA1* expression. There may be other targets of ERF1 which also participate this process since some difference in primary root length was observed between *ERF1ox asa1-1* and *asa1-1* (Fig 8D).

## Discussion

One of the best studied effects of ethylene on roots is the inhibition of root elongation[5,20]. A number of studies have indicated that ethylene inhibits root development through interaction with auxin. Ethylene has been shown to increase auxin synthesis, auxin transport to the elongation zone, and auxin signaling at the root tip[3,5–9,47,52]. ERF1, a downstream transcription factor in the ethylene signal pathway was reported to reduce primary root growth in the dark when constitutively expressed[27]. Until now, no detailed and explicit mechanism has been provided for its role in primary root elongation. In this study, we demonstrated that ERF1 directly regulates the expression of *ASA1*, a key enzyme in Trp biosynthesis where auxin is derived and known to play an important role in ethylene-regulated root development[8]. This work elaborates the mechanism by which the transcription factor ERF1 participates in primary root development and directly mediates crosstalk between ethylene and auxin biosynthesis during root elongation.

Through analyses of root response to ethylene with overexpression and knockdown lines of *ERF1* (Fig 2A–2C), we found that the length of the primary root was closely correlated to *ERF1* expression. These results imply that *ERF1* is involved in ethylene-mediated root elongation.

ERFs belong to a large gene family. Only a few ERF mutants show obvious phenotypes, probably due to functional redundancy. However, *ERF1* knockdown lines displayed longer root under normal and added ACC conditions (Fig 2B, 2C and 2G), indicating that ERF1 plays an important role in ethylene-inhibited root elongation. Furthermore, ERF1 controls primary root elongation by reducing cell elongation, but not cell division (Fig 2D–2F), which is consistent with ethylene signal pathway mutants[6].

Ethylene upregulates auxin biosynthesis in *Arabidopsis* seedlings to enhance inhibition of root elongation[6]. High auxin levels are known to reduce root growth[3]. Some auxin biosynthesis genes are ethylene responsive, and if mutated, cause some defects in root growth in the presence of ACC[8,9]. To understand how ERF1 mediates ethylene signaling in primary root elongation and particularly auxin biosynthesis, we analysed the promoters of all genes which participate in auxin biosynthesis, and found that two genes including *ASA1* and *YUCCA2* contained a GCC-box which can be specifically bound by ERF1. A recent study showed that ERF109, another member of the ERF family that is highly responsive to JA signaling, directly regulates both *ASA1* and *YUC2* and mediates crosstalk between JA and auxin biosynthesis[42]. The primary root elongation of the *yucca2* mutant, in the presence of added ACC, did not differ from wildtype. Considering that *ASA1* is ethylene responsive, *asa1* is ethylene insensitive in root-elongation[8], and there is a GCC-box in the promoter of *ASA1* which could be bound by ERF1, we hypothesized that *ASA1* might be a direct target of ERF1.

To confirm our hypothesis, we conducted *in vitro* binding (EMSA), yeast-one-hybrid, and chromatin immunoprecipitation (ChIP) experiments and confirmed that ERF1 could directly bind a conserved GCC-box element in the promoter of *ASA1 in vitro* and *in vivo* (Fig 4). We further confirmed our hypothesis by analyzing *ASA1pro*:*GUS* in *ERF1* knockdown and overexpression background. As we expected, the expression of *ASA1* was remarkably increased in *ERF1* overexpression background but reduced in the knockdown lines (Fig 5). Upon ACC treatment, the staining of *ASA1pro*:*GUS* in *ERF1* knockdown background becomes darker but still relatively weaker than that in Col-0 background at the root tip and DZ (Fig 5C), indicating that the ethylene responsiveness was not completely removed. In the *ERF1* knockdown lines, the induction of *ASA1* by ethylene was reduced but some induction was still retained compared to the wildtype (S4C Fig), which is consistent with the primary root phenotype (Fig 6). This may be due to incomplete suppression of *ERF1* by RNAi technique, alternatively, there may be additional components involved in this process. For instance, EIN3 was suggested to directly regulate *ASA1* based on the data of EIN3 ChIP-Seq experiments [63].

Meanwhile, analyses of *ASA1pro*:*GUS* reporter in ethylene signal pathway mutation background (*ein2-5*, *ein3-1*, *ctr1-1*, *EIN3ox*) showed that when ethylene signal pathway was enhanced, the expression of *ASA1* was also enhanced. Conversely, if ethylene signal pathway was blocked, the expression of *ASA1* was reduced and the induction by ACC was also impaired (Fig 7). These results explicated that *ASA1* is downstream of these ethylene signal pathway components.

Taken together, our results support a model in which ethylene stimulates auxin biosynthesis in roots through ethylene-responsive transcription factor ERF1 that positively regulates *ASA1*. As a consequence of activating *ASA1* expression, ERF1 increases the accumulation of auxin, which in turn decreases root elongation and alters root architecture.

## Methods

### Plant growth conditions and material

Surface-sterilised *Arabidopsis* seeds were treated for 10 min in 10% bleach and cold-treated for 3–4 d at 4°C, and grown on Murashige and Skoog (MS, 1 x salts) medium with 1% sucrose for the indicated days (see Figure legends). Seedlings were then transferred to plates supplemented with or without ACC for the indicated days, and the plates were placed vertically in a growth room. ACC (Sigma-Aldrich) was dissolved in water and prepared as a 2 mM stock solution. *Arabidopsis thaliana* 7-d-old seedlings were transferred to soil and grown to maturity in a growth room. All plants were grown under long-day conditions (16-h light / 8-h dark) at 22–24°C. Every experiment was repeated at least three times.

*Arabidopsis thaliana* ecotype Columbia-0 (Col-0) was used. Some plant materials used in this study were previously described: *ein2-5*[26], *ein3-1*[41], *ein3-1 eil1-1*[52], *ctr1-1*[25], *EIN3-FLAG* (*iE*/*qm*) (*EIN3ox*) [64], *ASA1*_*pro*_:*GUS* [8], *DR5*:*GUS*[65].

Plants were obtained from transformation of Col-0 plants with the *ERF1*_*pro*_:*GUS* construct, which contained a 3.0-kb promoter sequence with primers (S1 Table). *ERF1* over-expressing plants (*ERF1ox* #2, #6, and #12) were obtained from transformation of Col-0 with the *35S*_*pro*_:*ERF1* construct, which contains full-length *ERF1* (*At3g23240*). *ERF1* knockdown plants were obtained from transformation of Col-0 with the *35S*_*pro*_:*ERF1*:*RNAi* construct. *35S*:*HA-ERF1* were obtained from transformation of Col-0 plants with a vector comprised of a 35S promoter and HA sequence in front of the full-length *ERF1* cDNA. *35S*_*pro*_:*ERF1-GFP* transgenic lines were constructed in Col-0 *Arabidopsis*. *ERF1ox* transgenic plants used in genetic analysis of crossing *ERF1ox* with *asa1* were obtained from transformation of Col-0 plants with the vector comprised of an estradiol-inducible promoter in front of the full-length *ERF1*.

### DNA constructs and plant transformation

To prepare the *ERF1*_pro_:GUS construct, the primer set *ERF1pro*-*F* and *ERF1pro*-*R* (S1 Table), was used to amplify a 3.0 kb sequence from the *ERF1* promoter region. This was cloned into the binary vector pCB308R. To prepare the *ERF1* over-expression construct, the primer set, *ERF1*-*F* and *ERF1*-*R* and full-length *ERF1* fragments were amplified and cloned into the binary vector pCB2004[66]. To prepare the *ERF1* knock-down construct, the primer set *ERF1RNAiF* and *ERF1RNAiR* was used to amplify a 0.18 kb sequence from the ERF1 coding region, and cloned into the binary vector pCB2004B. To prepare the *HA*:*ERF1* over-expression construct, the primer set *ERF1HAF* and *ERF1HAR* was used to amplify the *HA*-*ERF1* coding sequence, and cloned into the binary vector pCB2004. To prepare the *35S*_*pro*_:*ERF1-GFP* construct, the product amplified by *ERF1GFPF* and *ERF1GFPR* was cloned into the binary vector pGWB5[67]. All of these constructs utilised the gateway system technology. To generate the *ERF1ox* transgenic plants with estradiol-inducible promoter, the PCR product amplified by pER8-*ERF1 F* and pER8-*ERF1 R* primers was cloned into the vector pER8 for transformation[68].These constructs were then individually transformed into *Agrobacterium tumefaciens* Strain (C58C1), and introduced into *Arabidopsis* plants by the floral dip method[69]. More than 30 transgenic lines were obtained for each construct.

### Histochemical GUS staining and confocal microscopy analysis

Histochemical staining for GUS activity in transgenic plants was conducted as previous described[70]. Seedlings were grown on MS medium as above, and stained in fresh GUS staining solution (1 mg/mL X-glucuronide in 0.1 M potassium phosphate, pH 7.2, 0.5 mM ferrocyanide, 0.5 mM ferricyanide, and 0.1% Triton X-100) at 37°C in the dark for the indicated time. After incubation, seedlings were cleared with a series of ethanol solutions (100%, 50% and 30%) and photographed. Images were captured using an OLYMPUS IX81 microscope and HiROX (Japan) MX5040RZ. For *in planta* GFP analysis, seedlings were stained in 10 mg/mL propidium iodide for 8 min and washed twice in water. Propidium iodide fluorescence and GFP were imaged under a ZEISS710 confocal laser scanning microscope: 488-nm and 543-nm lines of the laser were used for excitation, and emission was detected at 510 nm and 620 nm, respectively. More than 15 seedlings were examined for each transgenic line, and at least three independent experiments were displayed.

### Real-time qRT-PCR analysis

Total RNA was extracted with TRIzol reagent (Invitrogen) from seedlings or root samples. cDNA was prepared by TransScript RT kit (Invitrogen) for RNA reverse transcription, and was used for real-time quantitative RT-PCR. All quantitative RT-PCR assays were performed with a SYBR Premix Ex Taq II kit on StepOne real-time PCR system (Applied Biosystems) according to the manufacturer’s instructions. Expression levels of target genes were normalized to *Arabidopsis UBQ5*. All qRT-PCR experiments were performed at least three biological replicates. The primers used in this study have been listed in S1 Table.

### IAA content measurement

The free total IAA content was measured by ELISA as described[71].

### Yeast-one-hybrid assay

The procedures of the yeast one-hybrid assay were displayed as described previously with minor modifications[72]. A DNA fragment encoding *ERF1* was amplified with the primers *ERF1*_*Y1H*_ *F* and *ERF1*_*Y1H*_ *R*, and cloned into pAD-GAL4-2.1 (AD vector) to produce the *pAD/ERF1* plasmid. Two complementary core 30-bp DNA single strands including GCC-box in the promoter of *ASA1*, named *ASA1*_*Y1H*_ *F* and *ASA1*_*Y1H*_ *R*, were annealed and cloned into the reporter plasmid via *Sac*I and *Mlu*I restriction sites, pHIS2 (BD vector), which contained the nutritional reporter gene, *HIS3*.Two complementary DNA strands with three copies of the DNA sequence GCC-box, named *GCC*_*Y1H*_ *F* and *GCC*_*Y1H*_ *R* were annealed and cloned into plasmid *pHIS2* through *Sac*I and *Mlu*I restriction sites as a positive control. The *pAD/ERF1* construct containing the ERF1 cDNA sequence and the *pHIS2* reporter construct containing the GCC-box *cis*-element were co-transformed into Y187 yeast cells. For the negative control, the *pAD/ERF1* plasmid and the *pHIS2* empty plasmid were co-transformed into Y187 yeast cells. Yeast was cultured in SD/–Trp–Leu medium and then transferred to SD/–Trp–Leu–His medium containing10 mM 3-aminotriazole (Sigma) with dilutions as indicated. The plates were incubated at 30°Cfor 4 d and the results were observed. Growth of yeast cells on the SD/–Trp–Leu–His medium in the presence of 10 mM 3-aminotriazole indicated that the transcription factor could bind this *cis*-element, and activated relevant gene expression.

### ChIP-PCR assay

One gram each of six-day-old *35S*:*HA-ERF1*seedlings and Col-0 plants were harvested for ChIP experiments. The procedures were conducted essentially as previously described[73]. The enrichment of DNA fragments in the *ASA1* promoter was amplified by PCR using the following primer pairs: *ChIP-ASA1pro F*, *ChIP-ASA1pro R*, and *β-tubulin8* was used as negative control. The resultant PCR-products were resolved by electrophoresis on 2% agarose gels. The results displayed above represent at least three independent repeats.

To analyse the binding quantitatively, a qRT-PCR assay was performed on the basis of the procedure described previously[74]. The relative quantity value is presented as the DNA binding ratio. The same primers for the above PCR analysis were used for qRT-PCR.

### Electrophoretic mobility shift assay

The fragment of the full-length *ERF1* coding sequence was amplified by PCR with primers *ERF1*_*EMSA*_ *F* and *ERF1*_*EMSA*_ *R*, and cloned into the pMAL-C2 vector via *Eco*RI and *Xba*I restriction sites, to construct a plasmid for the expression of recombinant MYC2 protein in *Escherichia coli*.

Five individual synthetic 30-bp single-stranded DNA molecules containing the GCC-box were used, namely *ASA1*_*EMSA*_ *F-DIG*, *ASA1*_*EMSA*_ *R*, *ASA1*_*EMSA*_ *F*, *ASA1*_*EMSA-Mu*_ *F* and *ASA1*_*EMSA-Mu*_ *R*. These DNA fragments were annealed with their complementary oligonucleotides, *ASA1*_*EMSA*_ *F-DIG* with *ASA1*_*EMSA*_ *R* for EMSA labelled probe, *ASA1*_*EMSA*_ *R* with *ASA1*_*EMSA*_ *F* for EMSA competitive probe, *ASA1*_*EMSA-Mu*_ *F* with *ASA1*_*EMSA-Mu*_ *R* for EMSA non-competitive probe. EMSA was performed according to a DIG Gel Shift kit, 2^nd^ Generation (Roche).

## Supporting Information

S1 Fig Expression pattern of *ERF1* revealed by *ERF1pro*:*GUS* and subcellular localization of ERF1. (**a-c**) Seeds of *ERF1*_*pro*_:*GUS* were germinated on MS medium and stained for GUS expression. *ERF1* expression pattern in 4- (**a**), 6- (**b**), and 15-day-old (**c**) plants. Scale bar, 0.5 cm. (**d**) Twenty-five-day-old *ERF1*_*pro*_:*GUS* transgenic seedlings grown in soil. (**e**) Stem, siliques and flowers of 35-day-old *ERF1*_*pro*_:*GUS* transgenic lines grown in soil. (**f**) The cellular localization of ERF1 protein revealed by the *35S*_*pro*_:*ERF1-GFP* transgenic plants. The roots of 5-day-old *35S*_*pro*_:*ERF1-GFP* transgenic plants were photographed using a ZEISS710 confocal laser scanning microscope. Scale bar = 50 μm.
(DOC)

S2 Fig Primary root phenotype and the relative *ERF1* expression level in *ERF1* knockdown and overexpression lines compared to wildtype. (**a**) Seeds of the transgenic lines and wildtype were germinated vertically on MS medium for 5 days, and the representative seedlings were photographed. Scale bar, 1 cm. (**b**) The primary root length of the transgenic lines and wildtype was measured from 4 to 8 days. Data shown are average and SD (n = 20, *P<0.05, **P<0.01, ***P<0.001. Asterisks indicate Student’s t-test significant differences). (**c**) The expression level of *ERF1* in these materials was tested by qRT-PCR. Values are mean ± SD of three replicates (*P<0.05, ***P<0.001. Asterisks indicate Student’s t-test significant differences).
(DOC)

S3 Fig Root elongation of *ERF1* knockdown and overexpression lines of etiolated seedlings. (a) Images of representative 5-d-old etiolated seedlings grown in the MS medium are displayed. Genotypes are as indicated. Scale bar, 1 cm.(b) The primary root length of 5-d-old etiolated seedlings was measured. Data shown are average and SD (*P<0.05, ***P<0.001. Asterisks indicate Student’s t-test significant differences).
(DOC)

S4 Fig Root growth response of ethylene signalling mutants to ACC treatment and expression of *ERF1* and *ASA1* in *ERF1* knockdown lines. (**a**) The primary root elongation phenotype in the mutants as indicated. Ethylene signalling pathway-related mutants *ein2-5*, *ein3-1*, *ein3-1eil1*, *ctr1-1*, and wildtype (Col-0) were geminated on MS medium with 0 or 0.8 μM ACC for 5 d. The seeds of *EIN3ox* (*EIN3-FLAG* (*iE*/*qm*)) were grown on medium containing 1 μM β-estradiol and 0 or 0.8 μM ACC for 5 d, respectively. Scale bar, 1cm. (**b-c**) *ERF1* and *ASA1* expression levels in *ERF1* knockdown lines (*RNAi-1*, *RNAi-2*) and wildtype seedlings with or without 0.2 μM ACC treatment from 5 to 8 d. Total RNA was extracted from roots. The expression level of *ERF1* and *ASA1* in these materials was detected by qRT-PCR. Values are mean ± SD of three replicates.
(DOC)

S5 Fig The *ERF1* expression level in *ERF1* knockdown and overexpression lines. The expression level of *ERF1* in 5-day-old Col-0 wildtype, *ERF1* knockdown (*RNAi-1*, *RNAi-2*) and overexpression (*ERF1ox #2*, *ERF1ox #6*, *ERF1ox #12*) seedlings in *DR5*:*GUS* (**a**) and *ASA1*_*pro*_:*GUS* (**b**) backgrounds was tested by qRT-PCR. Values are mean ± SD of three replicas (*P<0.05, ***P<0.001. Asterisks indicate Student’s t-test significant differences).
(DOC)

S6 Fig The root phenotype and *ERF1* expression level in wildtype and *35S*:*HA-ERF1*. (**a**) The root phenotype of 5-day-old Col-0 wildtype and *35S*:*HA-ERF1*. Scale bar, 0.5 cm. (**b**) The *ERF1* expression level in 5-day-old wildtype and *35S*:*HA-ERF1* seedlings. Values are mean ± SD of three replicas (***P<0.001. Asterisks indicate Student’s t-test significant differences).
(DOC)

S7 Fig Primary root elongation of mutants in response to ACC. (a) Images of representative 5-d-old seedlings grown in the presence of 0, 0.2, 0.5, 1, and 10 μM ACC are displayed. Genotypes are as indicated. Scale bar, 1 cm. (b) Relative root length of 5-d-old seedlings (Col-0, *RNAi-1*, *asa1*, *ein2-5*) grown in the presence of 0, 0.2, 0.5, 1, and 10 μM ACC. The response of each genotype to ACC was expressed as the percentage of the root length at a particular concentration of ACC with respect to the average length of root in the absence of ACC. Data shown are average and SD (*P<0.05, **P<0.01, ***P<0.001. Asterisks indicate Student’s t-test significant differences).
(DOC)

S8 Fig Primary root elongation of *asa1-1* mutant in response to ACC. (a) The primary root phenotypes of Col-0, *asa1-1*, *ERF1ox* and *ERF1ox asa1-1* seedlings grown on MS medium with either 0 or 1 μM ACC for 5 d. Scale bar, 1 cm. (b-c) Primary root length of Col-0, *asa1-1*, *ERF1ox* and *ERF1ox asa1-1* seedlings grown on MS medium with either 0 or 1 μM ACC were measured at the fifth days. Data shown are average and SD (Values are mean ± SD, n = 20).
(DOC)

S1 Table Primers used in this study (5'- to -3'). (DOC)
