# Correction: *Arabidopsis* ERF1 Mediates Cross-Talk between Ethylene and Auxin Biosynthesis during Primary Root Elongation by Regulating *ASA1* Expression

**DOI:** 10.1371/journal.pgen.1006076

**Published:** 2016-05-18

**Authors:** Jie-Li Mao, Zi-Qing Miao, Zhen Wang, Lin-Hui Yu, Xiao-Teng Cai, Cheng-Bin Xiang

Fig 1, Fig 4A and 4C, Fig 8C and 8D, S2 Fig and S8 Fig are incorrectly labeled. Please see the complete, correct figure captions here.

**Fig 1:**
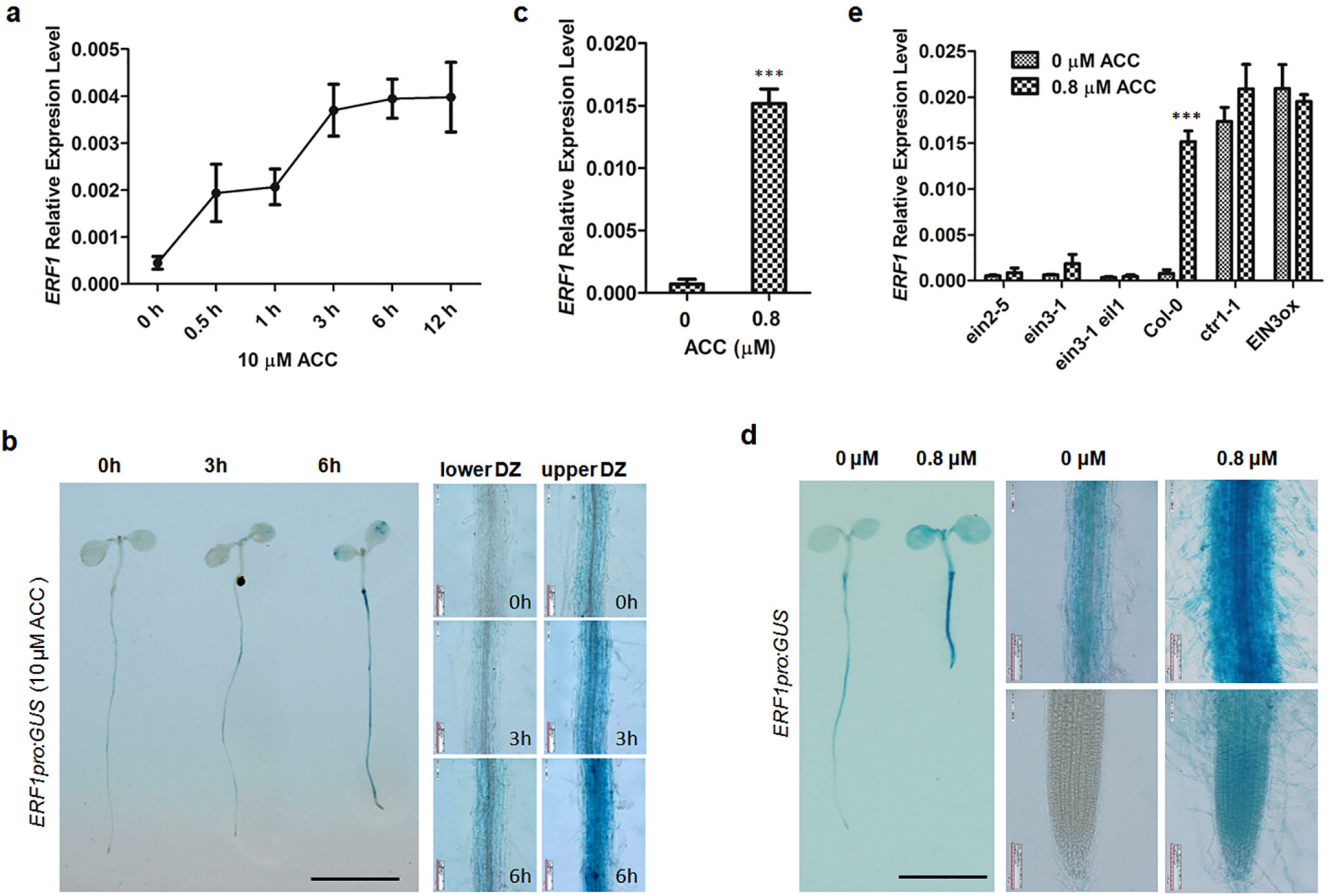
*ERF1* expression is responsive to ethylene. **(a)** Ethylene-induced *ERF1* expression in wildtype. Seeds of Col-0 were germinated on MS medium for 5 d then treated with 10 μM ACC for 0, 0.5, 1, 3, 6, and 12 h. The transcriptional level of *ERF1* was detected by quantitative RT-PCR (qRT-PCR). Values are mean ± SD of three replicates. ACC, 1-aminocyclopropane-1-carboxylic acid (precursor of ethylene biosynthesis). **(b)** Ethylene-induced expression of*ERF1pro*:*GUS*. Five-day-old seedlings of transgenic lines were treated with 10 μM ACC for 0, 3, and 6 h before GUS staining. Upper DZ and lower DZ represent different primary root regions. Scale bar, 0.5 cm. **(c)** Ethylene-induced expression of *ERF1* in wildtype. Seeds of Col-0 were germinated on MS medium with 0 or 0.8 μM ACC for 5 d, and relative *ERF1* transcription levels were measured by qRT-PCR. Values are mean ± SD of three replicates (***P<0.001). Asterisks indicate Student’s t-test significant differences. **(d)** Ethylene-activated expression in *ERF1pro*:*GUS* lines. Transgenic plants were grown on MS medium with either 0 or 0.8 μM ACC for 5 d before GUS staining assay. Scale bar, 0.5 cm. **(e)** The relative *ERF1* expression level was determined in ethylene signaling related mutants *ein2-5*, *ein3-1*, *ein3-1eil1*and compared to wildtype (Col-0) seedlings. Seedlings of constitutive (*ctr1-1*) and *β*-estradiol inducible *EIN3-FLAG (iE/qm)* (*EIN3ox*) expression were also examined. Seeds (*ein2-5*, *ein3-1*, *ein3-1eil1*,*ctr1-1*and Col-0) were geminated on MS medium with either 0 or 0.8 μM ACC for 5 d. Seeds of *EIN3ox* were grown on medium containing 1 μM β-estradiol and 0 or 0.8 μM ACC for 5 d. Roots of seedlings were used for qRT-PCR analysis. Values are mean ± SD of three replicas (***P<0.001). Asterisks indicate Student’s t-test significant differences.

**Fig 4:**
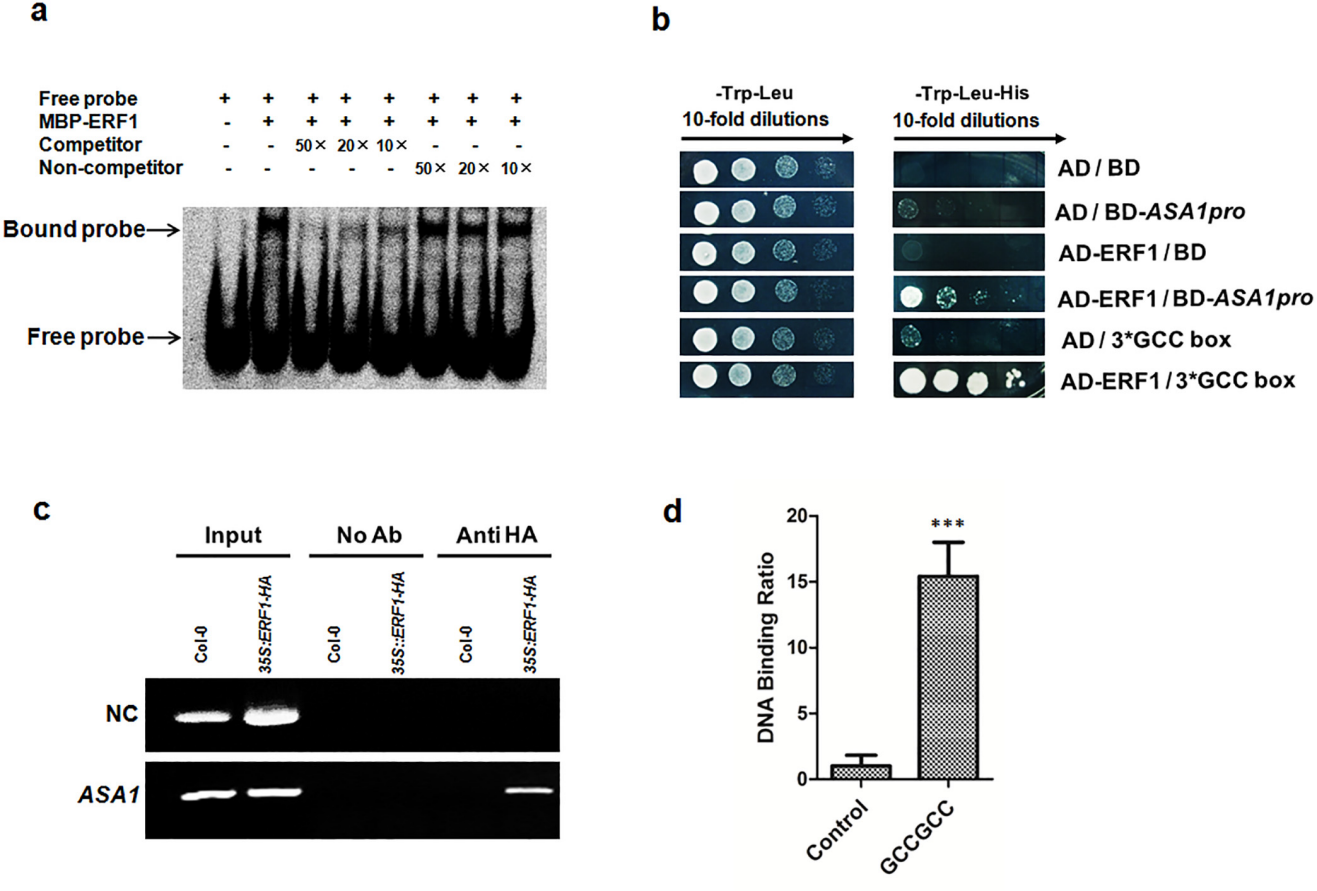
ERF1 directly binds to *ASA1* promoter region *in vitro* and *in vivo*. **(a)** EMSA assay for binding to GCC-box sequence in the promoter of *ASA1* byERF1 protein *in vitro*. Dig-labeled probes were incubated with ERF1-MBP protein. As indicated, unlabelled probes were used as competitors, unlabelled probes with mutated GCC-box sequence were used as non-competitors, and the ERF1-MBP protein bound probes were separated from free probes by an acrylamide gel. (**b**) Yeast-one-hybrid assay. pGADT7/ERF1 (AD-ERF1) and pHIS2/ASA1pro (BD-ASA1pro) constructs were co-transformed into yeast strain Y187. AD-empty and BD-empty, AD-empty and BD-ASA1pro, AD-ERF1 and BD-empty, AD-empty and BD-3*GCC-box were used as negative controls while AD-ERF1 and BD-3*GCC-box were used as a positive control. **(c)**. Chromatin immunoprecipitation-PCR for *ASA1* promoter. Roots of 5-day-old *35S*:*HA*:*ERF1* and Col-0 seedlings were used. Anti-HA antibodies were used for the enrichment of the DNA fragments containing GCC-box in the promoter of*ASA1*. The results were determined by PCR. *Tub8* was used as negative control (NC). (**d**) Quantitative real-time PCR was performed using the same ChIP products and PCR primers flanking GCC-boxes in *ASA1* promoter as in **c**. The region of *ASA1* that do not contain GCC-box was used as negative control. Values are mean ± SD of three replicas (***P<0.001). Asterisks indicate Student’s t-test significant differences.

**Fig 8:**
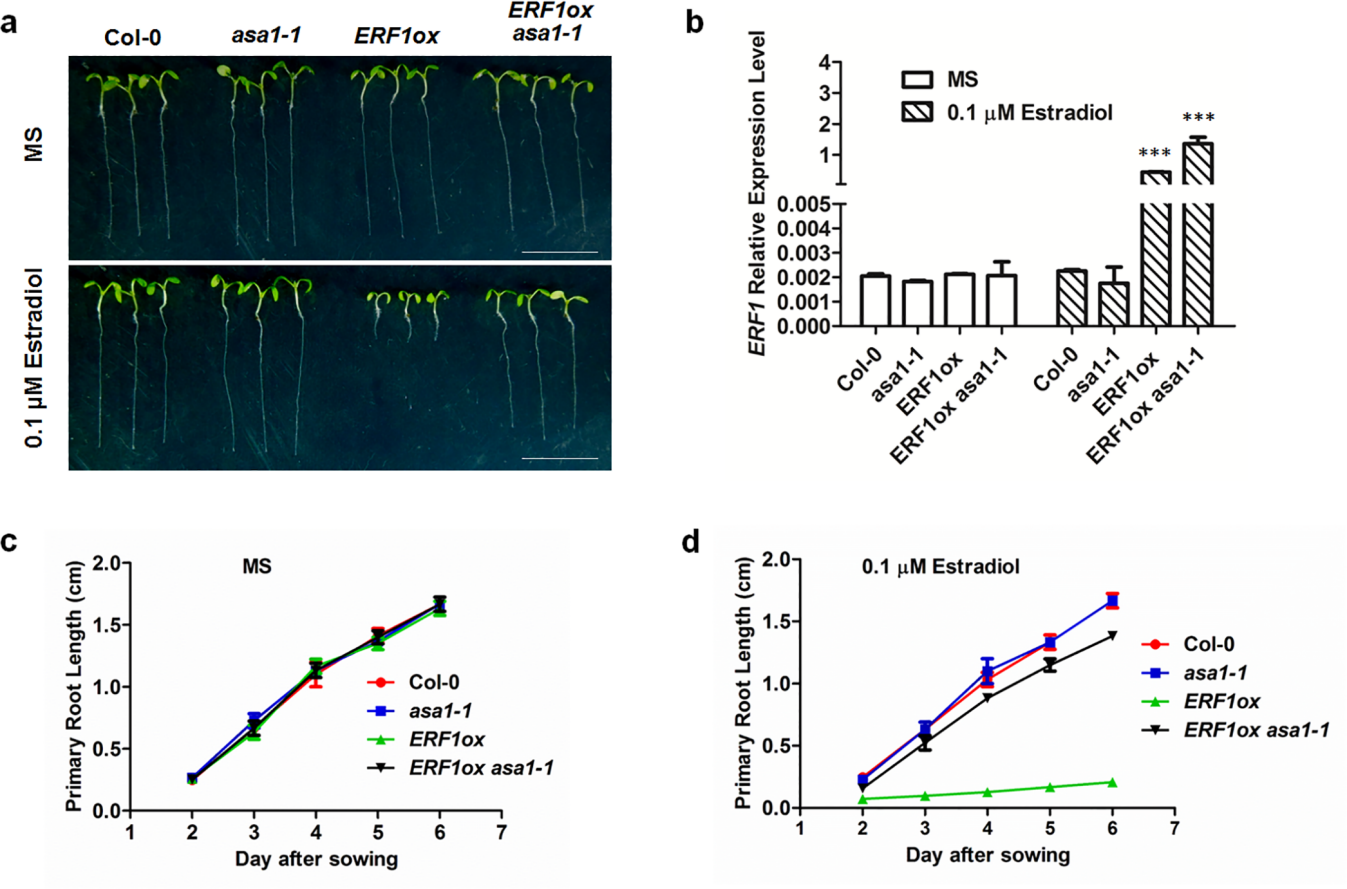
*ASA1* acts downstream of ERF1. **(a)** The primary root phenotypes of Col-0, *asa1-1*, *ERF1ox* and *ERF1ox asa1-1* seedlings grown on MS medium with either 0 or 0.1 μM estradiol for 5 d. *ERF1ox* is the transgenic plants expressing ERF1 protein under control of the estradiol-inducible promoter in Col-0 background. Scale bar, 1 cm. **(b)** qRT–PCR analysis of transcriptions of *ERF1*. The roots of 5-day-old Col-0, *asa1-1*, *ERF1ox* and *ERF1ox asa1-1* seedlings grown on MS medium with either 0 or 0.1 μM estradiol were used. Values are mean ± SD of three replicas (***P<0.001. Asterisks indicate Student’s t-test significant differences). **(c-d)**. Primary root length of Col-0, *asa1-1*, *ERF1ox* and *ERF1ox asa1-1* seedlings grown on MS medium with either 0 or 0.1μM estradiol were measured. Data shown are average and SD (Values are mean ± SD, n = 20).

## Supporting Information

S2 Fig Primary root phenotype and the relative *ERF1* expression level in *ERF1* knockdown and overexpression lines compared to wildtype. (**a**) Seeds of the transgenic lines and wildtype were germinated vertically on MS medium for 5 days, and the representative seedlings were photographed. Scale bar, 1 cm. (**b**) The primary root length of the 5-d-old transgenic lines and wildtype was measured. Data shown are average and SD (n = 20, *P<0.05, **P<0.01, ***P<0.001. Asterisks indicate Student’s t-test significant differences). (**c**) The expression level of *ERF1* in these materials was tested by qRT-PCR. Values are mean ± SD of three replicates (*P<0.05, ***P<0.001. Asterisks indicate Student’s t-test significant differences).
(DOC)

S8 Fig Primary root elongation of *asa1-1* mutant in response to ACC. **(a)** The primary root phenotypes of Col-0, *asa1-1*, *ERF1ox* and *ERF1ox asa1-1* seedlings grown on MS medium with either 0 or 1 μM ACC for 5 d. Scale bar, 1 cm. **(b-c)** Primary root length of Col-0, *asa1-1*, *ERF1ox* and *ERF1ox asa1-1* seedlings grown on MS medium with either 0 or 1 μM ACC were measured. Data shown are average and SD (Values are mean ± SD, n = 20).
(DOC)
