# Supplementary material for: Arabidopsis ERF1 Mediates Cross-Talk between Ethylene and Auxin Biosynthesis during Primary Root Elongation by Regulating ASA1 Expression
Source: PLoS Genet. 2016 Jan 8;12(1):e1005760. doi: 10.1371/journal.pgen.1005760 (PMC4706318; doi:10.1371/journal.pgen.1005760)
Supplement: S1 Fig — (a-c) Seeds of ERF1pro:GUS were germinated on MS medium and stained for GUS expression. ERF1 expression pattern in 4- (a), 6- (b), and 15-day-old (c) plants. Scale bar, 0.5 cm. (d) Twenty-five-day-old ERF1pro:GUS transgenic seedlings grown in soil. (e) Stem, siliques and flowers of 35-day-old ERF1pro:GUS transgenic lines grown in soil. (f) The cellular localization of ERF1 protein revealed by the 35Spro:ERF1-GFP transgenic plants. The roots of 5-day-old 35Spro:ERF1-GFP transgenic plants were photographed using a ZEISS710 confocal laser scanning microscope. Scale bar = 50 μm. (DOC) [file pgen.1005760.s001.doc]

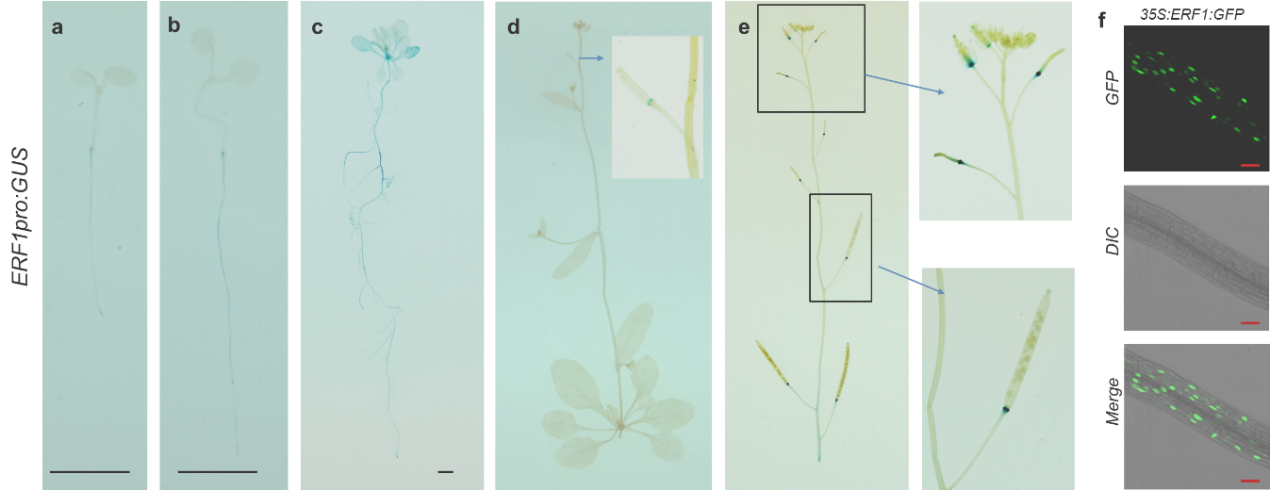


**S1 Fig. Expression pattern of *ERF1* revealed by *ERF1pro:GUS* and subcellular localization of ERF1.**

(**a-c**) Seeds of *ERF1_pro_:GUS* were germinated on MS medium and stained for GUS expression. *ERF1* expression pattern in 4- (**a**), 6- (**b**), and 15-day-old (**c**) plants. Scale bar, 0.5 cm. (**d**) Twenty-five-day-old *ERF1_pro_:GUS* transgenic seedlings grown in soil. (**e**) Stem, siliques and flowers of 35-day-old *ERF1_pro_:GUS* transgenic lines grown in soil. (**f**) The cellular localization of ERF1 protein revealed by the *35S_pro_:ERF1-GFP* transgenic plants. The roots of 5-day-old *35S_pro_:ERF1-GFP* transgenic plants were photographed using a ZEISS710 confocal laser scanning microscope. Scale bar=50 μm.
