# Supplementary material for: Arabidopsis ERF1 Mediates Cross-Talk between Ethylene and Auxin Biosynthesis during Primary Root Elongation by Regulating ASA1 Expression
Source: PLoS Genet. 2016 Jan 8;12(1):e1005760. doi: 10.1371/journal.pgen.1005760 (PMC4706318; doi:10.1371/journal.pgen.1005760)
Supplement: S2 Fig — (a) Seeds of the transgenic lines and wildtype were germinated vertically on MS medium for 5 days, and the representative seedlings were photographed. Scale bar, 1 cm. (b) The primary root length of the transgenic lines and wildtype was measured from 4 to 8 days. Data shown are average and SD (n = 20, *P<0.05, **P<0.01, ***P<0.001. Asterisks indicate Student’s t-test significant differences). (c) The expression level of ERF1 in these materials was tested by qRT-PCR. Values are mean ± SD of three replicates (*P<0.05, ***P<0.001. Asterisks indicate Student’s t-test significant differences). (DOC) [file pgen.1005760.s002.doc]

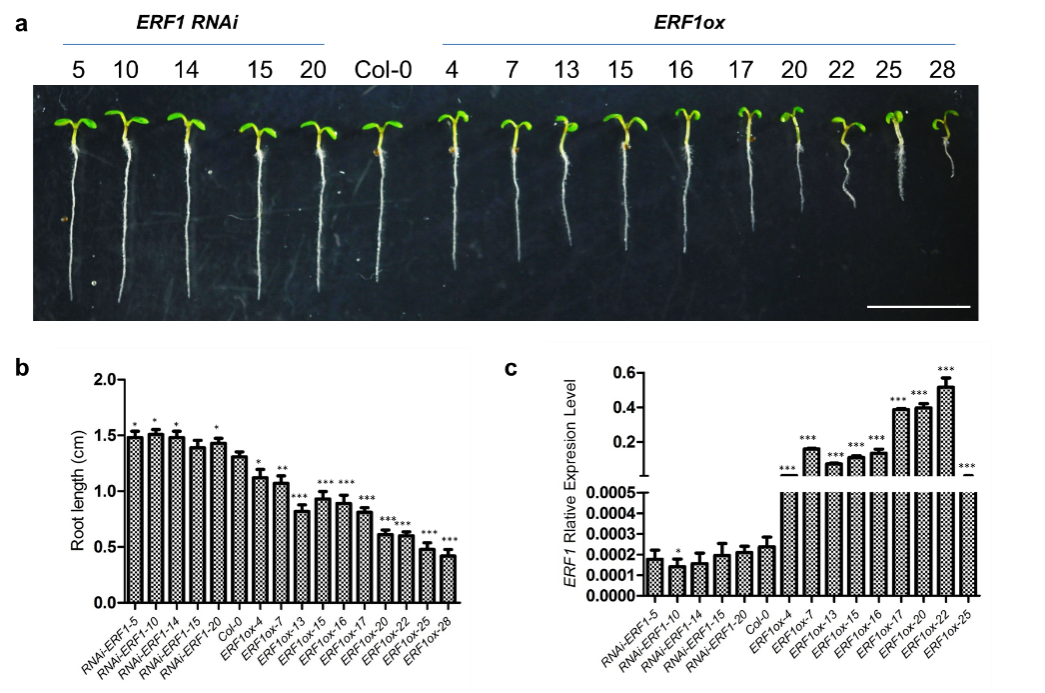


**S2 Fig. Primary root phenotype and the relative *ERF1* expression level in *ERF1* knockdown and overexpression lines compared to wildtype.**

(**a**) Seeds of the transgenic lines and wildtype were germinated vertically on MS medium for 5 days, and the representative seedlings were photographed. Scale bar, 1 cm. (**b**) The primary root length of the transgenic lines and wildtype was measured from 4 to 8 days. Data shown are average and SD (n = 20, **P*<0.05, ***P*<0.01, ****P*<0.001. Asterisks indicate Student’s t-test signiﬁcant differences). (**c**) The expression level of *ERF1* in these materials was tested by qRT-PCR. Values are mean ± SD of three replicates (**P*<0.05, ****P*<0.001. Asterisks indicate Student’s t-test signiﬁcant differences).
