# Supplementary material for: Arabidopsis ERF1 Mediates Cross-Talk between Ethylene and Auxin Biosynthesis during Primary Root Elongation by Regulating ASA1 Expression
Source: PLoS Genet. 2016 Jan 8;12(1):e1005760. doi: 10.1371/journal.pgen.1005760 (PMC4706318; doi:10.1371/journal.pgen.1005760)
Supplement: S3 Fig — (a) Images of representative 5-d-old etiolated seedlings grown in the MS medium are displayed. Genotypes are as indicated. Scale bar, 1 cm.(b) The primary root length of 5-d-old etiolated seedlings was measured. Data shown are average and SD (*P<0.05, ***P<0.001. Asterisks indicate Student’s t-test significant differences). (DOC) [file pgen.1005760.s003.doc]

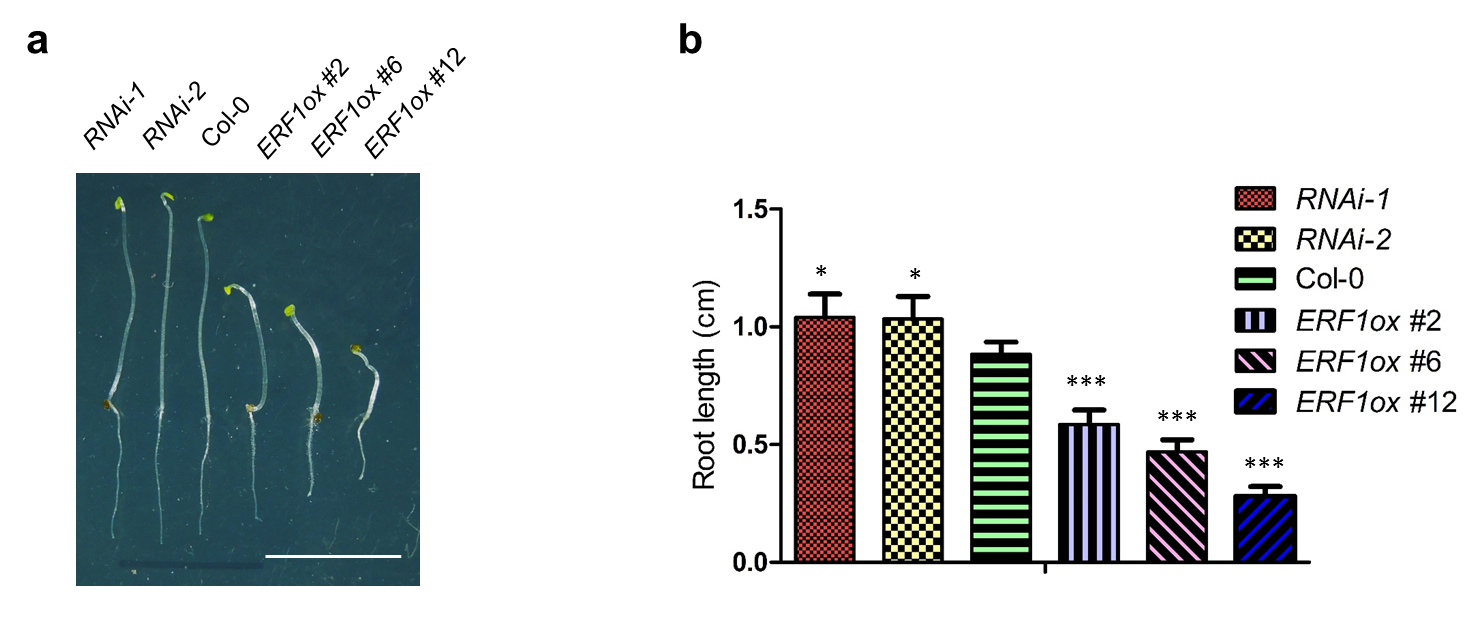


**S3 Fig. Root elongation of *ERF1* knockdown and overexpression lines of etiolated seedlings.**

(a) Images of representative 5-d-old etiolated seedlings grown in the MS medium are displayed. Genotypes are as indicated. Scale bar, 1 cm.

(b) The primary root length of 5-d-old etiolated seedlings was measured. Data shown are average and SD (*P<0.05, ***P<0.001. Asterisks indicate Student’s t-test significant differences).
