# Supplementary material for: Arabidopsis ERF1 Mediates Cross-Talk between Ethylene and Auxin Biosynthesis during Primary Root Elongation by Regulating ASA1 Expression
Source: PLoS Genet. 2016 Jan 8;12(1):e1005760. doi: 10.1371/journal.pgen.1005760 (PMC4706318; doi:10.1371/journal.pgen.1005760)
Supplement: S4 Fig — (a) The primary root elongation phenotype in the mutants as indicated. Ethylene signalling pathway-related mutants ein2-5, ein3-1, ein3-1eil1, ctr1-1, and wildtype (Col-0) were geminated on MS medium with 0 or 0.8 μM ACC for 5 d. The seeds of EIN3ox (EIN3-FLAG (iE/qm)) were grown on medium containing 1 μM β-estradiol and 0 or 0.8 μM ACC for 5 d, respectively. Scale bar, 1cm. (b-c) ERF1 and ASA1 expression levels in ERF1 knockdown lines (RNAi-1, RNAi-2) and wildtype seedlings with or without 0.2 μM ACC treatment from 5 to 8 d. Total RNA was extracted from roots. The expression level of ERF1 and ASA1 in these materials was detected by qRT-PCR. Values are mean ± SD of three replicates. (DOC) [file pgen.1005760.s004.doc]

**
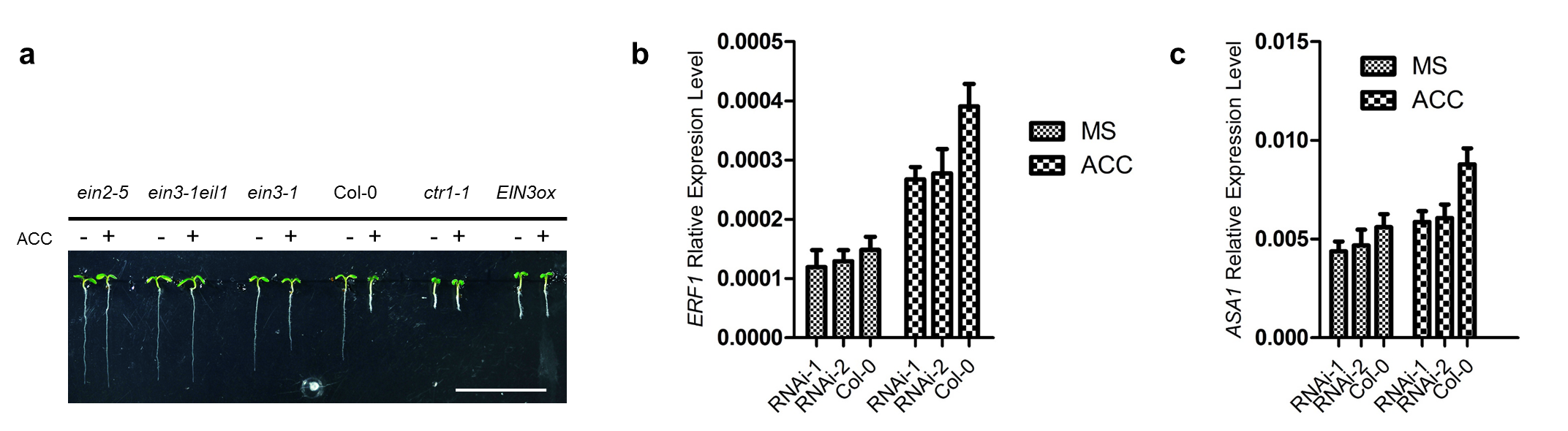
**

**S4 Fig. Root growth response of ethylene signalling mutants to ACC treatment and expression of *ERF1* and *ASA1* in *ERF1* knockdown lines.**

(**a**) The primary root elongation phenotype in the mutants as indicated. Ethylene signalling pathway-related mutants *ein2-5*, *ein3-1*, *ein3-1eil1*, *ctr1-1,* and wildtype (Col-0) were geminated on MS medium with 0 or 0.8 μM ACC for 5 d. The seeds of *EIN3ox* (*EIN3-FLAG* (*iE*/*qm*)) were grown on medium containing 1 μM β-estradiol and 0 or 0.8 μM ACC for 5 d, respectively. Scale bar, 1cm. (**b-c**) *ERF1* and *ASA1* expression levels in *ERF1* knockdown lines (*RNAi-1*, *RNAi-2*) and wildtype seedlings with or without 0.2 μM ACC treatment from 5 to 8 d. Total RNA was extracted from roots. The expression level of *ERF1* and *ASA1* in these materials was detected by qRT-PCR. Values are mean ± SD of three replicates.
