# Supplementary material for: Arabidopsis ERF1 Mediates Cross-Talk between Ethylene and Auxin Biosynthesis during Primary Root Elongation by Regulating ASA1 Expression
Source: PLoS Genet. 2016 Jan 8;12(1):e1005760. doi: 10.1371/journal.pgen.1005760 (PMC4706318; doi:10.1371/journal.pgen.1005760)
Supplement: S5 Fig — The expression level of ERF1 in 5-day-old Col-0 wildtype, ERF1 knockdown (RNAi-1, RNAi-2) and overexpression (ERF1ox #2, ERF1ox #6, ERF1ox #12) seedlings in DR5:GUS (a) and ASA1pro:GUS (b) backgrounds was tested by qRT-PCR. Values are mean ± SD of three replicas (*P<0.05, ***P<0.001. Asterisks indicate Student’s t-test significant differences). (DOC) [file pgen.1005760.s005.doc]

**
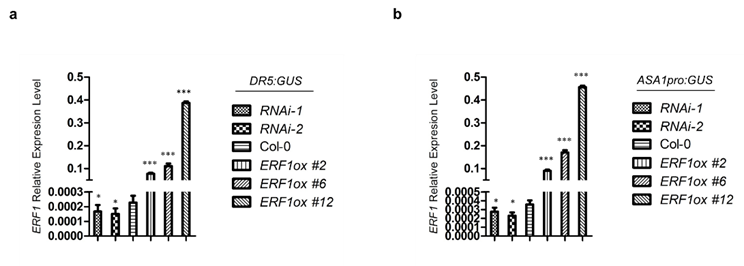
**

**S5 Fig. The *ERF1* expression level in *ERF1* knockdown and overexpression lines.**

The expression level of *ERF1* in 5-day-old Col-0 wildtype, *ERF1* knockdown (*RNAi-1*, *RNAi-2*) and overexpression (*ERF1ox #2*, *ERF1ox #6*, *ERF1ox #12*) seedlings in *DR5:GUS* (**a**) and *ASA1_pro_:GUS* (**b**) backgrounds was tested by qRT-PCR. Values are mean ± SD of three replicas (**P*<0.05, ****P*<0.001. Asterisks indicate Student’s t-test significant differences).
