# Supplementary material for: Arabidopsis ERF1 Mediates Cross-Talk between Ethylene and Auxin Biosynthesis during Primary Root Elongation by Regulating ASA1 Expression
Source: PLoS Genet. 2016 Jan 8;12(1):e1005760. doi: 10.1371/journal.pgen.1005760 (PMC4706318; doi:10.1371/journal.pgen.1005760)
Supplement: S6 Fig — (a) The root phenotype of 5-day-old Col-0 wildtype and 35S:HA-ERF1. Scale bar, 0.5 cm. (b) The ERF1 expression level in 5-day-old wildtype and 35S:HA-ERF1 seedlings. Values are mean ± SD of three replicas (***P<0.001. Asterisks indicate Student’s t-test significant differences). (DOC) [file pgen.1005760.s006.doc]

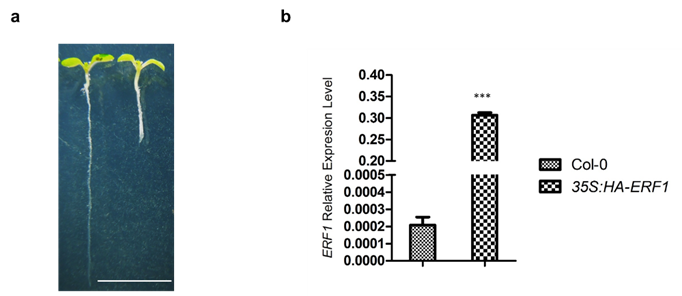


**S6 Fig. The root phenotype and *ERF1* expression level in wildtype and *35S:HA-ERF1*.**

(**a**) The root phenotype of 5-day-old Col-0 wildtype and *35S:HA-ERF1.* Scale bar, 0.5 cm.

(**b**) The *ERF1* expression level in 5-day-old wildtype and *35S:HA-ERF1* seedlings*.* Values are mean ± SD of three replicas (****P*<0.001. Asterisks indicate Student’s t-test significant differences).
