# Supplementary material for: Arabidopsis ERF1 Mediates Cross-Talk between Ethylene and Auxin Biosynthesis during Primary Root Elongation by Regulating ASA1 Expression
Source: PLoS Genet. 2016 Jan 8;12(1):e1005760. doi: 10.1371/journal.pgen.1005760 (PMC4706318; doi:10.1371/journal.pgen.1005760)
Supplement: S7 Fig — (a) Images of representative 5-d-old seedlings grown in the presence of 0, 0.2, 0.5, 1, and 10 μM ACC are displayed. Genotypes are as indicated. Scale bar, 1 cm. (b) Relative root length of 5-d-old seedlings (Col-0, RNAi-1, asa1, ein2-5) grown in the presence of 0, 0.2, 0.5, 1, and 10 μM ACC. The response of each genotype to ACC was expressed as the percentage of the root length at a particular concentration of ACC with respect to the average length of root in the absence of ACC. Data shown are average and SD (*P<0.05, **P<0.01, ***P<0.001. Asterisks indicate Student’s t-test significant differences). (DOC) [file pgen.1005760.s007.doc]

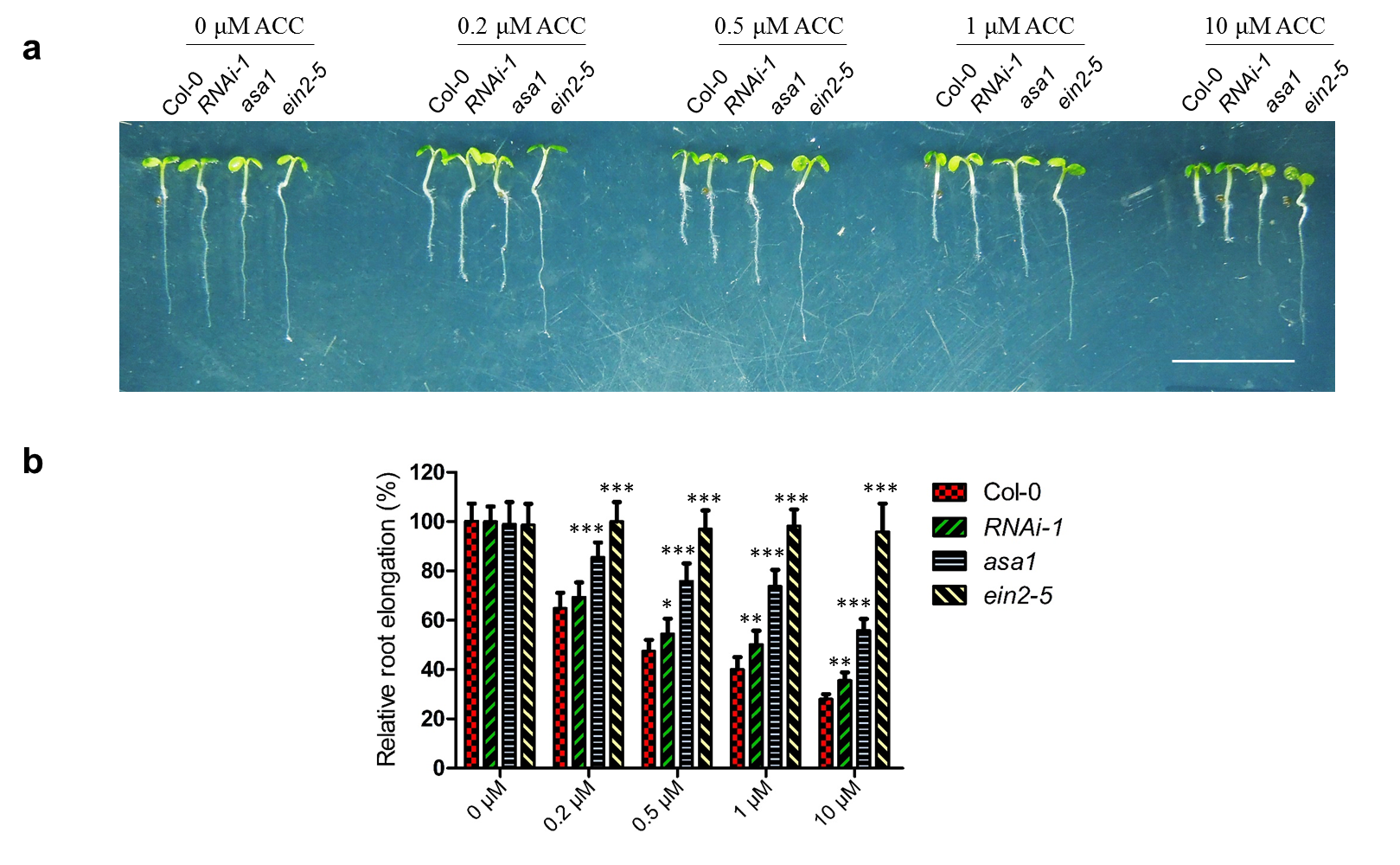


**S7 Fig. Primary root elongation of mutants in response to ACC.**

(a) Images of representative 5-d-old seedlings grown in the presence of 0, 0.2, 0.5, 1, and 10 μM ACC are displayed. Genotypes are as indicated. Scale bar, 1 cm.

(b) Relative root length of 5-d-old seedlings (Col-0, *RNAi-1*, *asa1*, *ein2-5*) grown in the presence of 0, 0.2, 0.5, 1, and 10 μM ACC. The response of each genotype to ACC was expressed as the percentage of the root length at a particular concentration of ACC with respect to the average length of root in the absence of ACC. Data shown are average and SD (*P<0.05, **P<0.01, ***P<0.001. Asterisks indicate Student’s t-test significant differences).
