# Supplementary material for: Arabidopsis ERF1 Mediates Cross-Talk between Ethylene and Auxin Biosynthesis during Primary Root Elongation by Regulating ASA1 Expression
Source: PLoS Genet. 2016 Jan 8;12(1):e1005760. doi: 10.1371/journal.pgen.1005760 (PMC4706318; doi:10.1371/journal.pgen.1005760)
Supplement: S8 Fig — (a) The primary root phenotypes of Col-0, asa1-1, ERF1ox and ERF1ox asa1-1 seedlings grown on MS medium with either 0 or 1 μM ACC for 5 d. Scale bar, 1 cm. (b-c) Primary root length of Col-0, asa1-1, ERF1ox and ERF1ox asa1-1 seedlings grown on MS medium with either 0 or 1 μM ACC were measured at the fifth days. Data shown are average and SD (Values are mean ± SD, n = 20). (DOC) [file pgen.1005760.s008.doc]

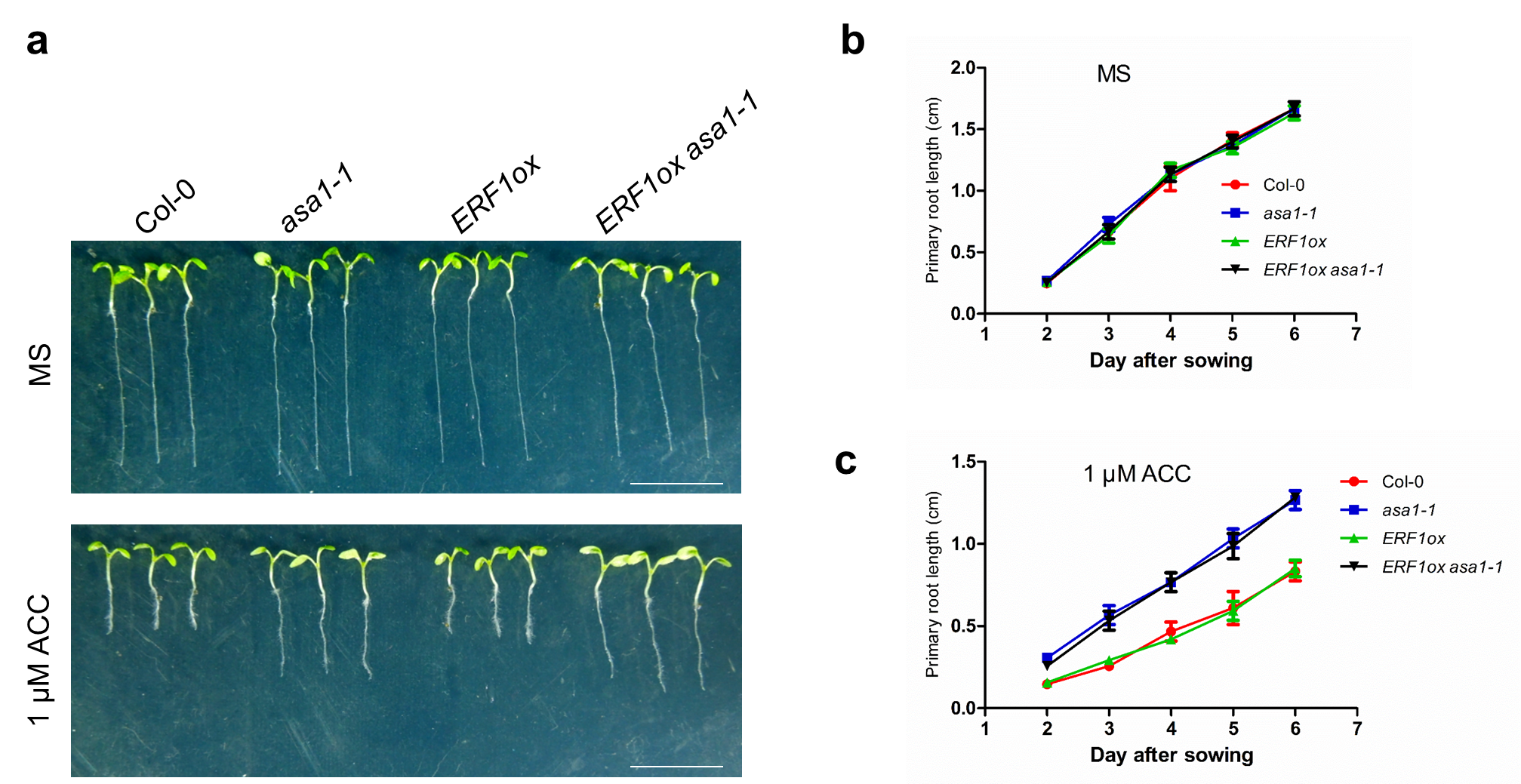


**S8 Fig. Primary root elongation of *asa1-1* mutant in response to ACC.**

(a) The primary root phenotypes of Col-0, *asa1-1*, *ERF1ox* and *ERF1ox asa1-1* seedlings grown on MS medium with either 0 or 1 μM ACC for 5 d. Scale bar, 1 cm. (b-c) Primary root length of Col-0, *asa1-1*, *ERF1ox* and *ERF1ox asa1-1* seedlings grown on MS medium with either 0 or 1 μM ACC were measured at the fifth days. Data shown are average and SD (Values are mean ± SD, n = 20).
