# Supplementary material for: Arabidopsis ERF1 Mediates Cross-Talk between Ethylene and Auxin Biosynthesis during Primary Root Elongation by Regulating ASA1 Expression
Source: PLoS Genet. 2016 Jan 8;12(1):e1005760. doi: 10.1371/journal.pgen.1005760 (PMC4706318; doi:10.1371/journal.pgen.1005760)
Supplement: S1 Table — (DOC) [file pgen.1005760.s009.doc]

**S1 Table. Primers used in this study (5'- to -3').**

| UBQ (Q-PCR) F | AGAAGATCAAGCACAAGCAT |
| --- | --- |
| UBQ (Q-PCR) R | CAGATCAAGCTTCAACTCCT |
| ERF1(Q-PCR) F | ATTCTTTCTCATCCTCTTCTTCT |
| ERF1(Q-PCR) R | CGAATCTCTTATCTCCGCCG |
| ASA1 (Q-PCR) F | ATGTCTTCCTCTATGAACGTAGC |
| ASA1 (Q-PCR) R | ACAGCGGTAAATTGGTATAAGG |
| IAA1 (Q-PCR) F | ACAATCCCAAGAAGAGCAATAAC |
| IAA1 (Q-PCR) R | CTCACTATACTTTAACGGAGAAG |
| IAA2 (Q-PCR) F | GAACAAGAACAAGAAGAAGAAGAAC |
| IAA2 (Q-PCR) R | GGGTAATCCAAGACATAGCTCT |
| ASA1 (ChIP-Q-PCR) F | GTTCGCTGCCTTCATGGTTC |
| ASA1 (ChIP-Q-PCR) R | TCCAGTAACGGTCACAGAAGA |
| NC (ChIP-Q-PCR) F | TTGTGCCCAAGAACAGAGGCT |
| NC (ChIP-Q-PCR) R | GAATCAAGTTTGGGAGAATAGCT |
| β-Tubulin8 F | CTTAAGCTCACCACTCCAAGCT |
| β-Tubulin8 R | GCACTTCCACTTCGTCTTCTTC |
| ERF1pro F | GGGGACAAGTTTGTACAAAAAAGCAGGCTGCAATGTGATAAAGAAGATAGTG |
| ERF1pro R | GGGGACCACTTTGTACAAGAAAGCTGGGTGTAGAAAAAATACTCTGTTTCTTG |
| ERF1 F | GGGGACAAGTTTGTACAAAAAAGCAGGCTATGGATCCATTTTTAATTCAGTCC |
| ERF1 R | GGGGACCACTTTGTACAAGAAAGCTGGGTTCACCAAGTCCCACTATTTTCA |
| ERF1_RNAi_ F | GGGGACAAGTTTGTACAAAAAAGCAGGCTATGGATCCATTTTTAATTCAGTCC |
| ERF1_RNAi_ R | GGGGACCACTTTGTACAAGAAAGCTGGGTTATAGGTTTGTTGCGTGGACTG |
| ERF1_HA_-F | GTTTGTACAAAAAAGCAGGCTATGTACCCATACGATGTTCCAGATTACGCTATGGATCCATTTTTAATTCAGTC |
| ERF1_HA_ R | CTTTGTACAAGAAAGCTGGGTTCACCAAGTCCCACTATTTTCA |
| ERF1GFP F | GGGGACAAGTTTGTACAAAAAAGCAGGCTATGGATCCATTTTTAATTCAGTC |
| ERF1GFP R | GGGGACCACTTTGTACAAGAAAGCTGGGTCCCAAGTCCCACTATTTTCAGAA |
| ERF1_Y1H_ F | CCGCTCGAGATGGATCCATTTTTAATTCAGTCC |
| ERF1_Y1H_ R | GCTCTAGATCACCAAGTCCCACTATTTTCAG |
| ASA1_Y1H_ F | CTCAAACATTCCGGCCGCCACCGAGTTCTTAA |
| ASA1_Y1H_ R | CGCGTTAAGAACTCGGTGGCGGCCGGAATGTTTGAGAGCT |
| GCC_Y1H_ F | CGCCGCCGCCGCCGCCGCCA |
| GCC_Y1H_ R | CGCGTGGCGGCGGCGGCGGCGGCGAGCT |
| ChIP-ASA1pro F | GTTCGCTGCCTTCATGGTTC |
| ChIP-ASA1pro R | TCCAGTAACGGTCACAGAAGA |
| ERF1_EMSA_ F | CGGAATTCATGGATCCATTTTTAATTCAGTC |
| ERF1_EMSA_ R | TGCTCTAGATCACCAAGTCCCACTATTTTC |
| ASA1_EMSA_ F-DIG | TCAAACATTCCGGCCGCCACCGAGTTCTTA |
| ASA1_EMSA_ R | TAAGAACTCGGTGGCGGCCGGAATGTTTGA |
| ASA1_EMSA_ F | TCAAACATTCCGGCCGCCACCGAGTTCTTA |
| ASA1_EMSA-Mu_ F | TCAAACATTCCGCATTGAACCGAGTTCTTA |
| ASA1_EMSA-Mu_ R | TAAGAACTCGGTTCAATGCGGAATGTTTGA |
| pER8-*ERF1 F* | CCG CTCGAGATGGATCCATTTTTAATTCAGTC |
| pER8-*ERF1 R* | GGACTAGTTCACCAAGTCCCACTATTTTCA |
